# Hypoxic Endothelial-Cell-Derived Exosomal lnc-DKK3 Reprograms Tumor-Associated Macrophages via USP47/PD-L1/RelA Axis to Promote Glioma Progression

**DOI:** 10.34133/research.1431

**Published:** 2026-09-14

**Authors:** Zanhan Wang, Yongbo Zhao, Fan Fan, Chenchen Ji, Yamiao Chen, Penghui Li, Ang Ji, Wei Bai, Hongqing Chen, Yunchao Yuan, Juan Li, Xiuli Cao, Xia Li, Jifeng Liu, Junlong Zhao, Sanzhong Li

**Affiliations:** ^1^ Department of Neurosurgery, Xijing Hospital, Fourth Military Medical University, Xi’an, China.; ^2^ Department of Neurosurgery, Xijing 986 Hospital, Fourth Military Medical University, Xi’an, China.; ^3^ State Key Laboratory of Holistic Integrative Management of Gastrointestinal Cancers, Department of Medical Genetics and Developmental Biology, Fourth Military Medical University, Xi’an, China.; ^4^ Department of Orthopedics, Xijing Hospital, Fourth Military Medical University, Xi’an, China.; ^5^ Department of Otolaryngology Head and Neck Surgery and Deep Underground Space Medical Center, West China Hospital, Sichuan University, Chengdu, China.

## Abstract

The hypoxic microenvironment in gliomas critically contributes to malignant progression. This study investigates the functional role and molecular mechanisms of long noncoding RNAs derived from hypoxic endothelial cell exosomes in reprogramming tumor-associated macrophages and driving glioma progression. We identified a novel long noncoding RNA, lnc-DKK3, enriched in exosomes secreted by hypoxic endothelial cells. Nuclear-localized lnc-DKK3 suppresses PD-L1 ubiquitination in macrophages by binding to USP47, promoting M2-like polarization and immunosuppression. Additionally, the lnc-DKK3/USP47 axis stabilizes RelA, augmenting its transcriptional activity and collectively accelerating glioma progression. Thus, hypoxic endothelial-cell-derived lnc-DKK3 drives M2 polarization via the USP47/PD-L1 pathway and promotes glioma progression via the USP47/RelA axis, highlighting its therapeutic potential. To translate these insights, we developed a macrophage-specific CRISPR interference–liquid nanoparticle platform targeting the lnc-DKK3 super-enhancer and demonstrated its synergistic sensitization to anti-PD-1 therapy in preclinical models.

## Introduction

Glioblastoma (GBM), the most aggressive primary brain tumor, is characterized by a profoundly immunosuppressive and hypoxic tumor microenvironment (TME) that drives therapeutic resistance and disease progression [1]. Hypoxic stress functions as a master regulator, fostering a niche that sustains glioma stem cells and promotes aberrant angiogenesis [2]. Vascular endothelial cells actively engage in bidirectional signaling with tumors. The microfluidic models demonstrate that endothelial cells can modulate oxygen gradients within tumor spheroids [3,4] and undergo phenotypic changes in response to hypoxic signals, particularly shaping TME through complex paracrine signaling [5–7]. This paracrine dialogue is critically mediated by exosomes, which serve as key vehicles for intercellular communication. Tumor- and stromal-cell-derived exosomes carry bioactive molecules to recipient cells, including proteins and noncoding RNAs (ncRNAs), to recipient cells [8,9], thereby remodeling the immune cells and facilitating the formation of pre-metastatic niches [10,11]. In tumors, exosomes from endothelial-cell-derived exosomes have been shown to deliver specific cargo that influences immune evasion, angiogenesis, metastasis, and chemoresistance [12,13]. However, the mechanisms by which hypoxic endothelial exosomal long noncoding RNAs (lncRNAs) orchestrate immunosuppression and glioma progression remain poorly defined [14].

Immunosuppression drives resistance to immune checkpoint inhibitors in GBM [15–17]. This immunosuppressive TME is dominated by tumor-associated macrophages (TAMs) polarized toward an M2-like, pro-tumoral phenotype [18] and is further attributed to the intrinsic immune-evasive properties of glioma stem cells (GSCs) [19]. TAMs repress cytotoxic T-cell function, facilitate the generation of myeloid-derived suppressor cells and regulatory T cells, and secrete factors that support tumor growth [20,21]. GSCs, meanwhile, contribute to therapeutic resistance and recurrence and are known to interact with and modulate their microenvironment [22,23]. Increasing evidence highlights those endothelial cells, via exosome-mediated delivery, actively regulate both TAM remodeling and GSCs self-renewal [24,25]. However, whether and how hypoxic niches specifically alter endothelial-derived exosomes to simultaneously modulate TAM-mediated immunosuppression and fortify GSC-driven immune evasion remains unclear [26]. Few studies have examined how hypoxic endothelial exosomal lncRNAs mediate the dual interaction between macrophages and glioma cells, leaving a critical gap in understanding how endothelial paracrine signaling coordinates immune reprogramming and tumor cell stemness.

Exosomal lncRNAs regulate gene expression at epigenetic, transcriptional, and posttranscriptional levels, presenting important potential as therapeutic targets. Nuclear-localized lncRNAs exert potent and sustained regulatory effects by directly engaging with chromatin and the transcriptional machinery [27,28], functioning as scaffolds, decoys, or guides to modulate gene expression [29]. Specific nuclear lncRNAs influence NF-κB signaling and the DNA damage response, thereby contributing to tumor aggressiveness [30,31]. In glioma, the nuclear lncRNA ST7-AS2 promotes tumorigenesis by regulating VEGFR2 transcription [32–34], while other lncRNAs control the epithelial–mesenchymal transition [35]. However, whether hypoxic endothelial exosomes deliver nucleus-localized lncRNAs that concurrently reprogram TAMs and modulate glioma stemness remains unexplored [36,37].

In this study, we identified ENST00000527132 (lnc-DKK3) from hypoxic endothelial exosomes as a core functional molecule. Exosomal lnc-DKK3 transcriptionally activates USP47 in TAMs and glioma cells, stabilizing both PD-L1 and the NF-κB subunit RelA. This axis promotes PD-L1 expression in TAMs to foster immunosuppression and enhances RelA-driven stemness in glioma cells. Employing a CRISPR interference (CRISPRi) tool to target the lnc-DKK3 enhancer effectively suppressed USP47 transcription, remodeling the immunosuppressive TME and sensitizing gliomas to anti-PD-1 therapy in vivo. Thus, we reveal that hypoxic endothelial exosomal lnc-DKK3 serves as a dual-function signaling hub that simultaneously orchestrates TAM immunosuppressive polarization via the USP47/PD-L1 axis and promotes glioma stemness via the USP47/RelA axis, establishing a cohesive mechanistic link between endothelial paracrine signaling and immune-stromal co-evolution. Our findings elucidate a cohesive hypoxia-driven signaling axis and propose a promising combinatory immunotherapeutic strategy for glioma by targeting the endothelial exosomal lnc-DKK3–USP47 pathway.

## Results

### Hypoxic endothelial-cell-derived exosomes promote glioma progression by remodeling the glioma microenvironment

**Fig. 1.**
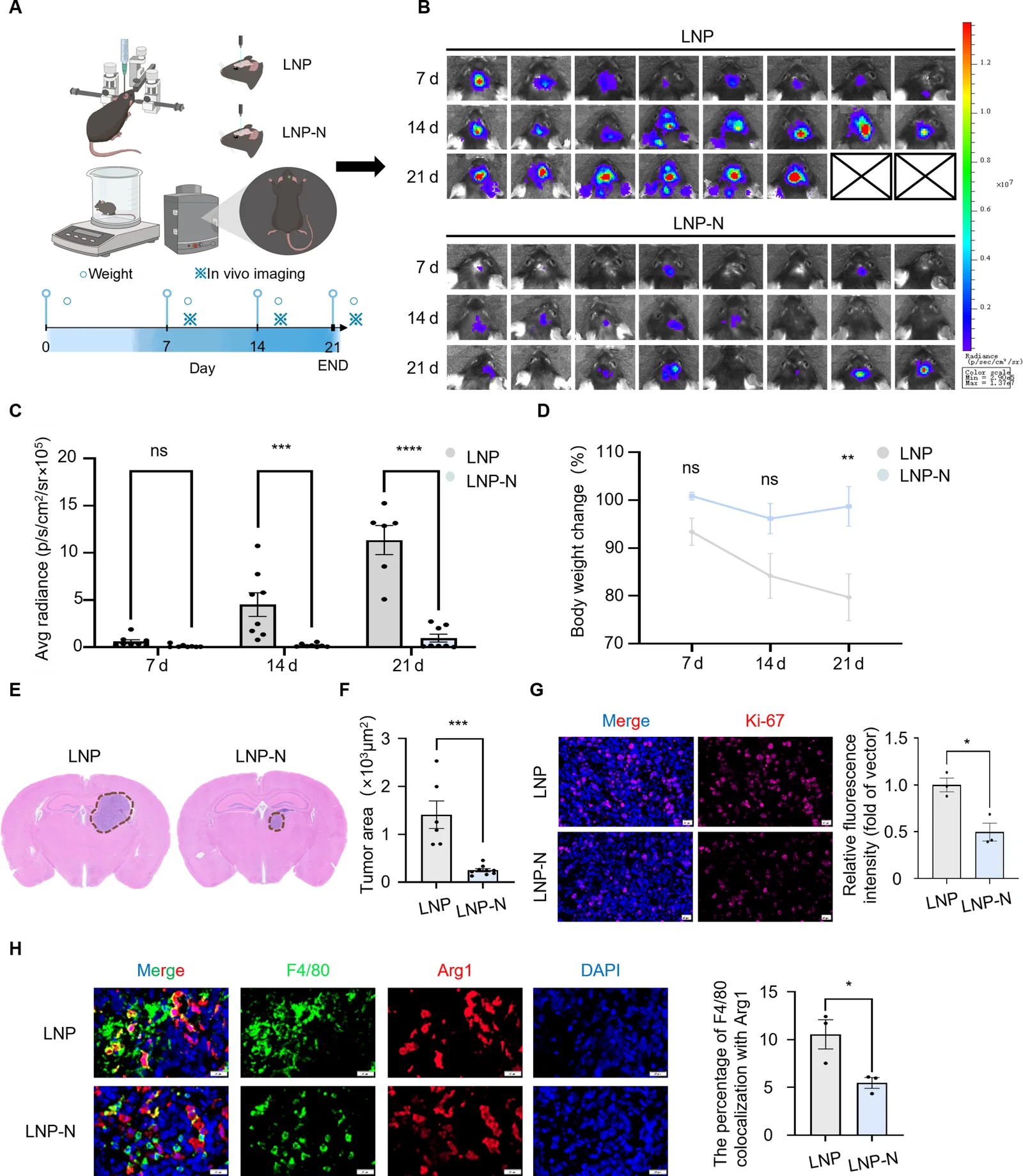
Blocking the endothelial exosome-mediated paracrine pathway suppresses glioblastoma progression in mice. (A) Schematic representation of the experimental design: an orthotopic glioma model with endothelial paracrine blockade using APRPG@Nexinhib20@LNP (LNP-N). (B and C) In vivo imaging demonstrated reduced intracranial tumor fluorescence in LNP-N-treated mice (*n* = 8). (D) Body weight remained stable throughout the treatment period. (E and F) Hematoxylin and eosin (HE) staining of brain sections at the experimental endpoint. (G) Immunofluorescence analysis revealed a lower Ki-67 (red) signal in the LNP-N group. (H) Reduced M2 macrophage markers in the LNP-N group indicated amelioration of the immunosuppressive microenvironment. *P < 0.05, **P < 0.01, ***P < 0.001, ****P < 0.0001; ns, not significant; 2-tailed unpaired Student *t* test; mean ± SEM; scale bar: 50 μm.

Given the pivotal role of endothelial-cell-derived exosomes in tumor progression, this study systematically investigated the impact of on the glioma microenvironment. Drawing upon prior literature, we constructed APRPG@Nexinhib20@LNP (APRPG@Nexinhib20@lipid nanoparticle, LNP-N) [38,39] encapsulating the Rab27a inhibitor Nexinhib20 [40] to specifically block endothelial exosome secretion (Fig. 1A). Prior to therapeutic evaluation, we validated the safety and targeting specificity of LNP-N; systemic administration resulted in no significant weight loss or organ abnormalities (Fig. S1A), and LNP-N was preferentially internalized by endothelial cells compared to immune or tumor cells (Fig. S1C), confirming its specificity. In a mouse GBM model, blocking endothelial exosome secretion using LNP-N significantly suppressed glioma outgrowth, as evidenced by markedly attenuated intracranial fluorescence signals (Fig. 1B and C). Mice in the LNP-N-treated group maintained stable body weight, in contrast to the tumor-induced weight loss observed in controls (Fig. 1D). Hematoxylin and eosin staining on day 21 revealed significantly smaller tumor areas in the LNP-N group compared to controls (Fig. 1E and F). Furthermore, Ki-67 expression was significantly reduced, indicating the inhibition of tumor cell proliferation (Fig. 1G). Staining analysis of tumor tissues from our mouse model revealed a significant reduction in M2 macrophage infiltration in the LNP-N-treated group (Fig. 1H). Together, these findings demonstrate that vascular endothelial cells in the hypoxic tumor microenvironment contribute to glioma proliferation and immune microenvironment remodeling through exosome-mediated paracrine signaling, and that targeted inhibition of this pathway effectively impedes disease progression.

To determine whether blocking endothelial exosome secretion influences brain exosome levels, we performed immunofluorescence staining for CD31 co-localized with the exosomal markers CD9 and TSG101. In LNP-N-treated mice, CD31^+^CD9^+^ and CD31^+^TSG101^+^ double-positive events were significantly reduced (Fig. S1B), confirming the effective blockade of endothelial exosome secretion and a decrease in exosome signals within the TME, consistent with the reduced tumor burden. Staining analysis also revealed a significant reduction in M2 macrophage infiltration in LNP-N-treated tumors (Fig. 1H).

Collectively, these findings demonstrate that vascular endothelial cells in the hypoxic TME contribute to tumor proliferation and immune remodeling through exosome-mediated paracrine signaling, and that targeted inhibition of this pathway effectively impedes disease progression.

### Lnc-DKK3 is a significantly up-regulated regulatory molecule in endothelial-cell-derived exosomes

**Fig. 2.**
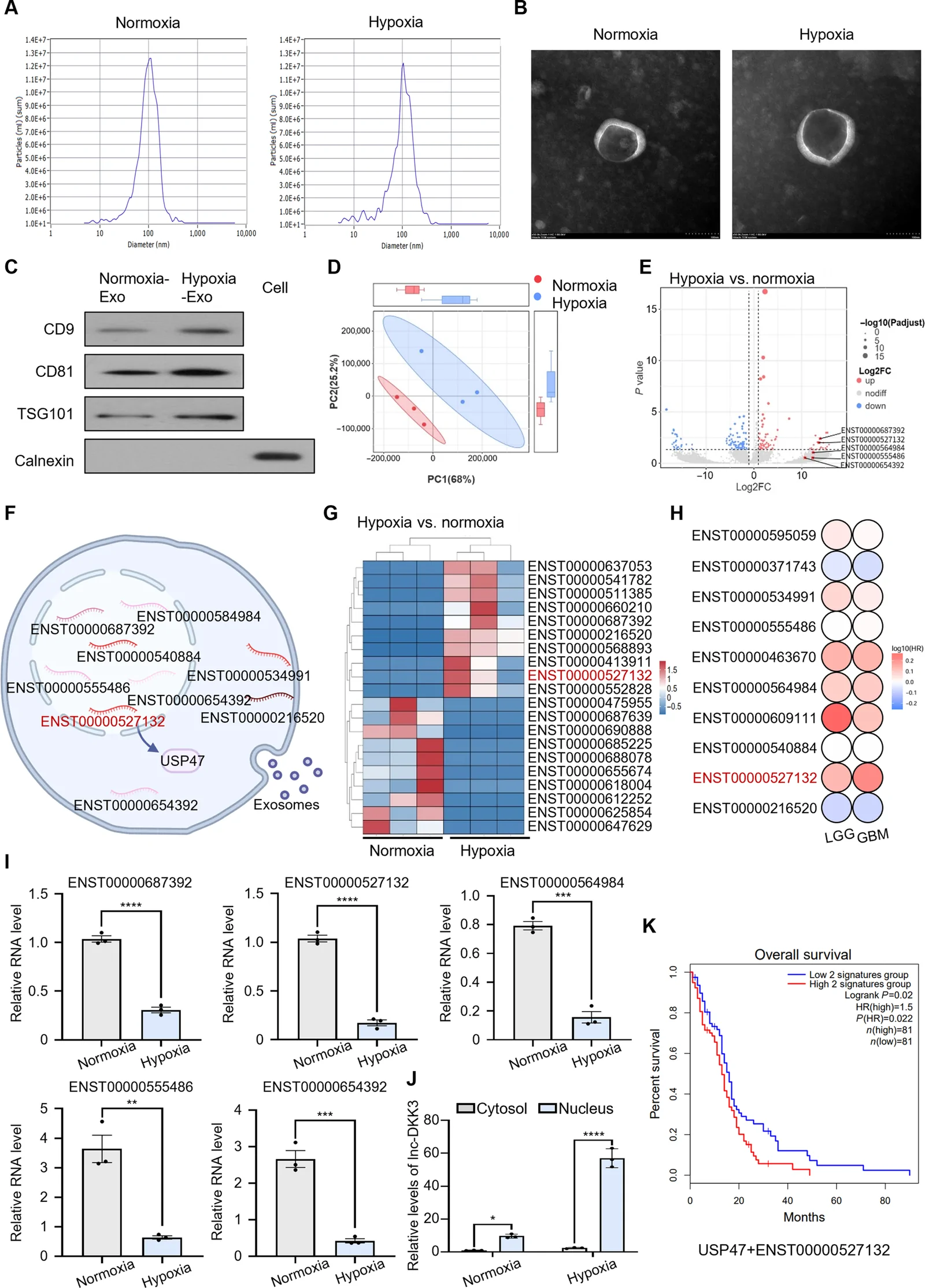
Screening for key long noncoding RNAs (lncRNAs) in hypoxic endothelial-cell-derived exosomes that regulate glioma. (A and B) Characterization of human-umbilical-vein-endothelial-cell-derived exosomes under hypoxic versus normoxic conditions: particle-size distribution (A) and mass spectrometry profiles (B). (C) Western blot analysis confirming exosomal markers. (D) Principal component analysis plot revealing distinct RNA expression profiles between groups. (E to H) Differential lncRNA analysis: volcano plot (E) highlighting up-regulated lncRNAs in hypoxia; subcellular localization (F); heatmaps of differentially expressed exosomal lncRNAs (G) and their correlation with overall survival in lower-grade glioma (LGG) and in patients with glioblastoma (GBM) (H). HR, hazard ratio. (I) Quantitative reverse transcription polymerase chain reaction (qRT-PCR) validation of the top 5 lncRNAs. (J) Nuclear/cytoplasmic fractionation confirming nuclear enrichment of lnc-DKK3. (K) Kaplan–Meier curve demonstrating that high co-expression of lnc-DKK3/USP47 correlates with poor survival in patients with TCGA GBM (log-rank *P* < 0.05). *P < 0.05, **P < 0.01, ***P < 0.001, ****P < 0.0001; 2-tailed unpaired Student *t* test or one-way analysis of variance (ANOVA); mean ± SEM.

To identify key factors within hypoxic endothelial-cell-derived exosomes that influence tumor progression, we performed high-throughput sequencing on exosomes isolated from human umbilical vein endothelial cells (HUVECs) cultured under normoxic and hypoxic conditions. Mass spectrometry and Western blot analyses confirmed hypoxia-induced alterations in exosomal cargo (Fig. 2A to C), while principal component analysis revealed distinct RNA expression profiles between the groups (Fig. 2D). Given that lncRNAs regulate gene expression at epigenetic, transcriptional, and posttranscriptional levels, we focused on screening differentially expressed lncRNAs. Volcano plot analysis identified significantly altered candidates (Fig. 2E). Since nucleus-localized lncRNAs can recruit chromatin-modifying complexes to exert potent regulatory effects, we examined the cellular localization of differentially expressed lncRNAs (Fig. 2F). A heatmap visually displays the top 10 significantly up-regulated and down-regulated lncRNAs in hypoxic exosomes (Fig. 2G), and bioinformatics analysis revealed correlations with glioma patient survival (Fig. 2H). We selected the top 5 nuclear-localized lncRNAs for quantitative reverse transcription polymerase chain reaction (qRT-PCR) validation (Fig. 2I). To experimentally verify the nuclear localization of lnc-DKK3, we performed nuclear/cytoplasmic fractionation followed by qRT-PCR. Under both normoxic and hypoxic conditions, lnc-DKK3 was enriched more than 10-fold in the nuclear fraction, with no significant difference in nuclear-to-cytoplasmic ratio between oxygen conditions, confirming its constitutive nuclear localization (Fig. 2J). Integrated bioinformatics analysis identified Ensembl ID ENST00000527132 as significantly highly expressed, with markedly higher levels in GBM versus low-grade glioma tissues (Fig. 2K). Synthesizing these multilayered screening results, we identified ENST00000527132 as the core candidate molecule within hypoxic endothelial exosomes mediating crosstalk with glioma cells and designated it lnc-DKK3.

### Lnc-DKK3 promotes glioma progression by regulating USP47 to influence macrophages

**Fig. 3.**
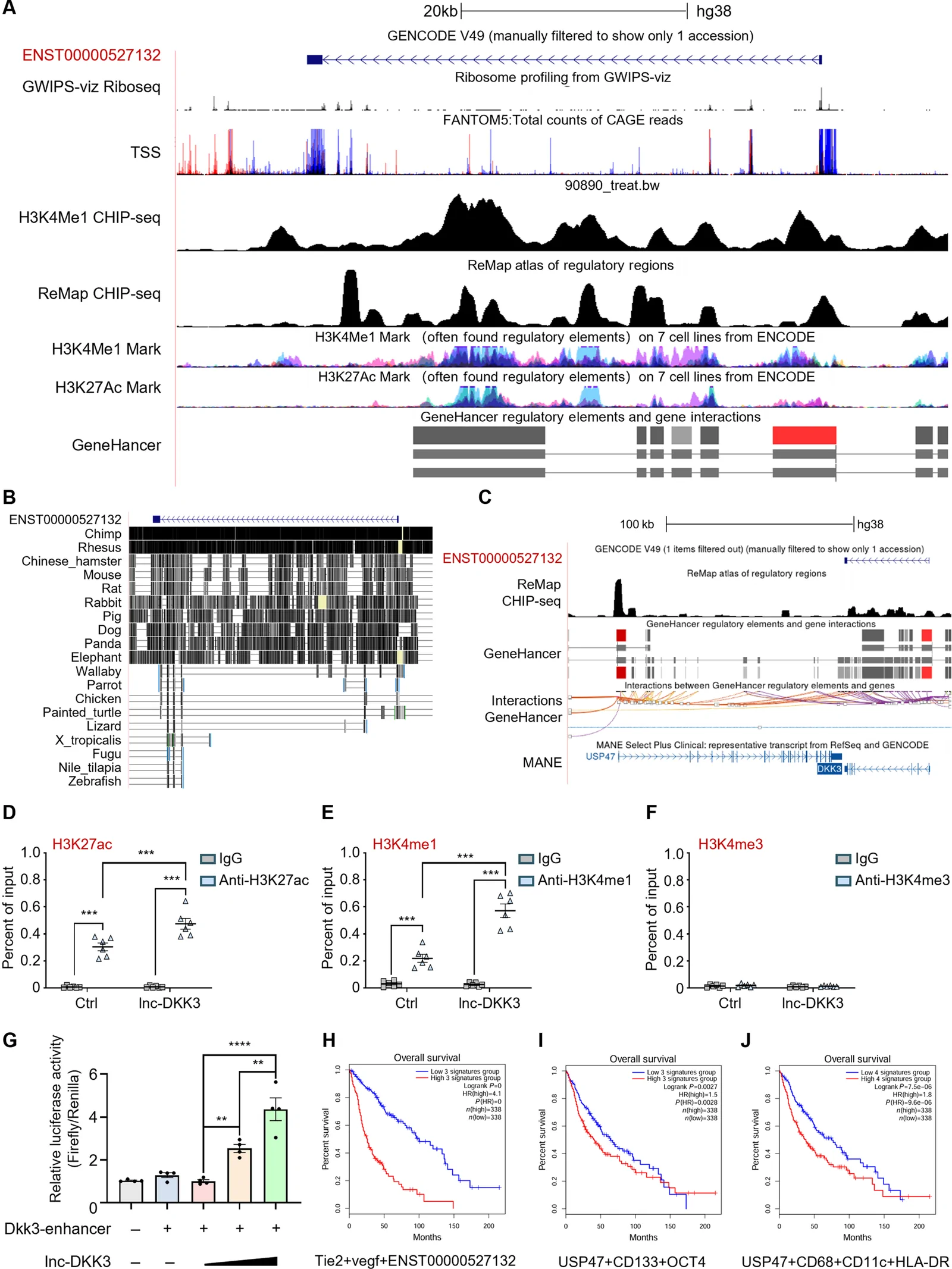
Molecular characterization and clinical prognostic value of lnc-DKK3. (A to C) Bioinformatic characterization of lnc-DKK3. (A) Analysis using Cistrome Data Browser analysis revealed the ChIP-seq peak distribution of lnc-DKK3 and USP47 at promoters of nucleotide synthesis genes. (B) Cross-species analysis identified conserved regions within lnc-DKK3. (C) Lnc-DKK3 regulates the enhancer of its upstream protein, USP47. (D to F) ChIP-qPCR for H3K4me1, H3K27Ac, and H3K4me3 demonstrated significantly increased active enhancer marks and H3K4me3 occupancy at the USP47 promoter in lnc-DKK3-expressing cells, confirming its active enhancer state. (G) Dual-luciferase assay indicated that the enhancer drives luciferase expression in a lnc-DKK3-dependent manner; mutation of the predicted binding motif abolished this effect. (H to J) Kaplan–Meier analysis of patients with TCGA glioma demonstrated that the co-expression of endothelial (Tie2+Vegf+ENST00000527132), stem cell (USP47+CD133+OCT4), and macrophage (USP47+CD68+CD11c+HLA-DR) signatures correlated with unfavorable survival (log-rank *P* < 0.05). **P < 0.01, ***P < 0.001, ****P < 0.0001; 2-tailed unpaired Student *t* test or one-way ANOVA; mean ± SEM.

To confirm that lnc-DKK3 possesses the molecular characteristics of a typical lncRNA and to assess its clinical significance, we performed a series of bioinformatic analyses. The University of California, Santa Cruz database revealed that the genomic locus of lnc-DKK3 overlaps with the DKK3 protein-coding region and contains a putative enhancer marked by H3K4me1 and H3K27Ac that may promote USP47 expression. Tracks including transcription start site (TSS), genome-wide information on protein synthesis (GWIPS) ribosome profiling, and cap analysis of gene expression (CAGE) reads are shown in this genome browser plot (Fig. 3A). Database analysis indicated low sequence conservation across species (Fig. 3B), and the Matched Annotation from NCBI and EBI (MANE) track suggested a regulatory interaction between lnc-DKK3 and the USP47 enhancer (Fig. 3C). To validate its noncoding nature, we assessed its protein-coding potential; lnc-DKK3 lacks a complete open reading frame and a canonical Kozak sequence, supporting its classification as a lncRNA (Fig. S2A).

To experimentally test the predicted enhancer–promoter interaction, we performed chromatin immunoprecipitation (ChIP)–qPCR using antibodies against H3K4me1, H3K27Ac, and H3K4me3. These assays confirmed the enrichment of active enhancer marks at the USP47 promoter in lnc-DKK3-expressing cells (Fig. 3D to F). To directly evaluate *cis*-regulatory function, we conducted dual-luciferase reporter assays. The enhancer sequence drove luciferase expression upon lnc-DKK3 co-expression, while mutation of the predicted binding motif abolished this effect (Fig. 3G).

To investigate clinical relevance, we performed survival analysis using public databases. Kaplan–Meier curves showed that high lnc-DKK3 expression in endothelial cells was significantly associated with shorter overall survival in patients with glioma (Fig. 3H). High USP47 expression in glioma stem cells (Fig. 3I) and macrophages was also correlated with poor survival (Fig. 3J), whereas USP47 expression in T cells, regulatory T cells, and B cells showed no significant prognostic correlation (Fig. S2B to D), suggesting cell-type-specific relevance of the lnc-DKK3/USP47 axis. These findings suggest that lnc-DKK3 may serve as a potential molecular marker for unfavorable prognosis in patients with glioma.

### Lnc-DKK3 promotes tumor progression by inducing M2 polarization of macrophages

**Fig. 4.**
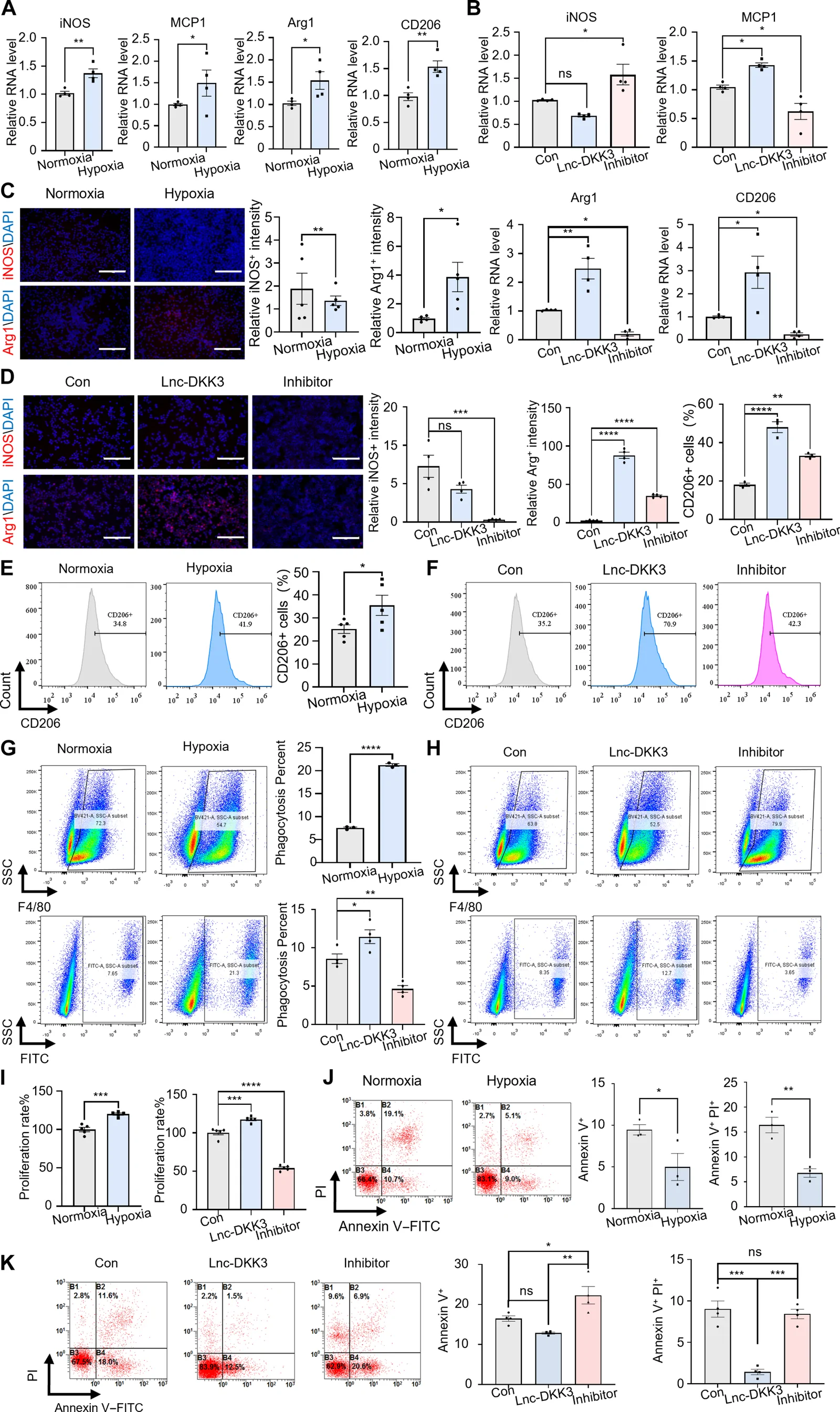
Lnc-DKK3 promotes tumor progression by inducing M2 polarization of macrophages. (A) qRT-PCR analysis of macrophage polarization marker expression following co-culture with conditioned medium from normoxic or hypoxic endothelial cells. (B) qRT-PCR analysis of polarization marker expression in macrophages overexpressing lnc-DKK3. (C) Immunofluorescence staining and quantification results. (D) Immunofluorescence staining and quantification. (E) Validation of polarization markers by flow cytometry and statistical analysis. (F) Validation of polarization markers by flow cytometry and statistical analysis. (G) Assessment of macrophage phagocytic capacity. (H) Assessment of macrophage phagocytic capacity. (I) Proliferation was enhanced in lnc-DKK3-overexpressing macrophages and effect reversed by USP47 inhibition. (J) Flow cytometric analysis and statistical quantification of macrophage apoptosis after co-culture with conditioned medium from normoxic or hypoxic endothelial cell. (K) Detection of macrophage apoptosis. *P < 0.05, **P < 0.01, ***P < 0.001, ****P < 0.0001; ns, not significant; 2-tailed unpaired Student *t* test or one-way ANOVA; mean ± SEM; scale bar: 150 μm.

Previous findings suggested that high lnc-DKK3 expression remodels the glioma TME, with differences observed in Arginase-1 (Arg1)^+^ macrophage infiltration. To elucidate the regulatory mechanism by which hypoxic endothelial cells regulate macrophage polarization, conditioned medium from normoxic and hypoxic HUVECs were collected and co-cultured with immortalized bone-marrow-derived macrophages (iBMDMs) for 24 h. qRT-PCR (Fig. 4A), immunofluorescence (Fig. 4C), and flow cytometry (Fig. 4E) confirmed that hypoxic endothelial conditioned medium significantly up-regulated M2 marker expression. To verify that exosomes, rather than soluble factors, mediate this effect, exosomes were isolated from normoxic and hypoxic HUVEC supernatants and used to treated iBMDMs directly. Hypoxic exosomes significantly up-regulated M2 markers (Arg1 and CD206) at the mRNA level, whereas M1 markers (inducible nitric oxide synthase [iNOS] and monocyte chemoattractant protein 1 [MCP1]) exhibited no significant changes; these results were corroborated by immunofluorescence analysis for iNOS and Arg1 (Fig. S3A to C). Furthermore, the phagocytic capacity (Fig. 4G), proliferative potential (Fig. 4I), and apoptotic potential (Fig. 4J) of macrophages were markedly enhanced following co-culture with tumor cells. To directly verify the role of lnc-DKK3, it was overexpressed in iBMDMs. Lnc-DKK3 overexpression recapitulated the effects of hypoxic conditioned medium, driving M2 polarization characterized by elevated M2 marker expression. Mechanistically, treatment of lnc-DKK3-overexpressing macrophages with a USP47-specific inhibitor [41] significantly suppressed M2 polarization (Fig. 4B, D, and F) and attenuated phagocytic capacity (Fig. 4H), proliferative potential (Fig. 4I), and apoptotic potential (Fig. 4K) following co-culture with tumor cells. In summary, hypoxic endothelial cells deliver lnc-DKK3 via exosomes, up-regulating USP47 in macrophages and driving immunosuppressive M2 polarization, thereby promoting glioma progression.

### Lnc-DKK3 promotes malignant behaviors and enhances stemness in glioma

**Fig. 5.**
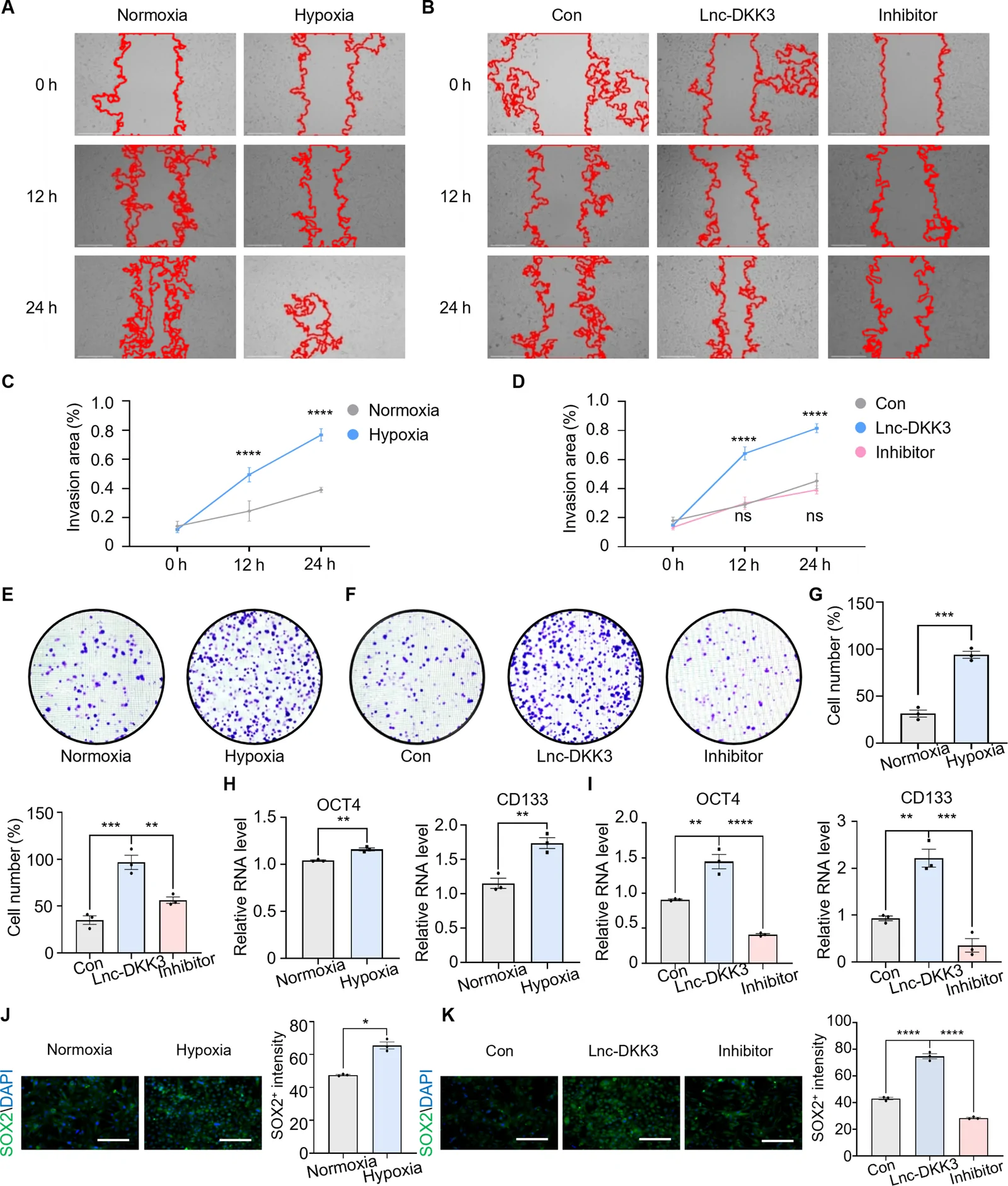
Lnc-DKK3 promotes malignant biological behaviors of glioma cells by regulating USP47. (A to D) Migration assays indicated that hypoxia and lnc-DKK3 overexpression promoted GL261 cell migration, whereas USP47 inhibition reversed this effect. (E to G) Proliferation assays demonstrated enhanced cell growth under hypoxia or lnc-DKK3 overexpression, which was attenuated by USP47 inhibition. (H and I) qRT-PCR analysis revealed up-regulated stemness markers (CD133 and OCT4) in lnc-DKK3 overexpressing cells, an effect reversed by USP47 inhibition. (J and K) Immunofluorescence confirmed increased SOX2 expression upon lnc-DKK3 overexpression, which was reduced by USP47 inhibition. *P < 0.05, **P < 0.01, ***P < 0.001, ****P < 0.0001; 2-tailed unpaired Student *t* test or one-way ANOVA; mean ± SEM; scale bar: 150 μm.

To investigate the biological function of lnc-DKK3 in glioma progression, we performed overexpression and functional rescue experiments in GL261 cells. High expression of lnc-DKK3 significantly enhanced the malignant phenotype of glioma cells. Cell migration assays demonstrated that hypoxic endothelial conditioned medium or lnc-DKK3 overexpression markedly increased GL261 migratory ability, whereas USP47 inhibition attenuated this effect (Fig. 5A to D). Similarly, colony formation assays revealed that hypoxic conditioned medium or lnc-DKK3 overexpression significantly enhanced glioma cell proliferation, which was partially reversed by USP47 inhibitor treatment (Fig. 5E to G). qRT-PCR analysis showed that stemness-related genes (CD133 and OCT4) were significantly up-regulated in GL261 cells treated with hypoxic conditioned medium or overexpressing lnc-DKK3, suggesting enhanced self-renewal and tumorigenic potential (Fig. 5H and I). To corroborate these findings at the protein level, we performed immunofluorescence staining for SOX2. Consistent with the mRNA results, SOX2 protein expression was markedly elevated in GL261 cells treated with hypoxic conditioned medium or overexpressing lnc-DKK3, while USP47 inhibition effectively attenuated this up-regulation (Fig. 5J and K). Collectively, these results indicate that lnc-DKK3 promotes glioma cell proliferation, migration, and stemness maintenance by up-regulating USP47, thereby driving malignant tumor progression.

### Lnc-DKK3 promotes macrophage phenotype remodeling by up-regulating PD-L1 via USP47

**Fig. 6.**
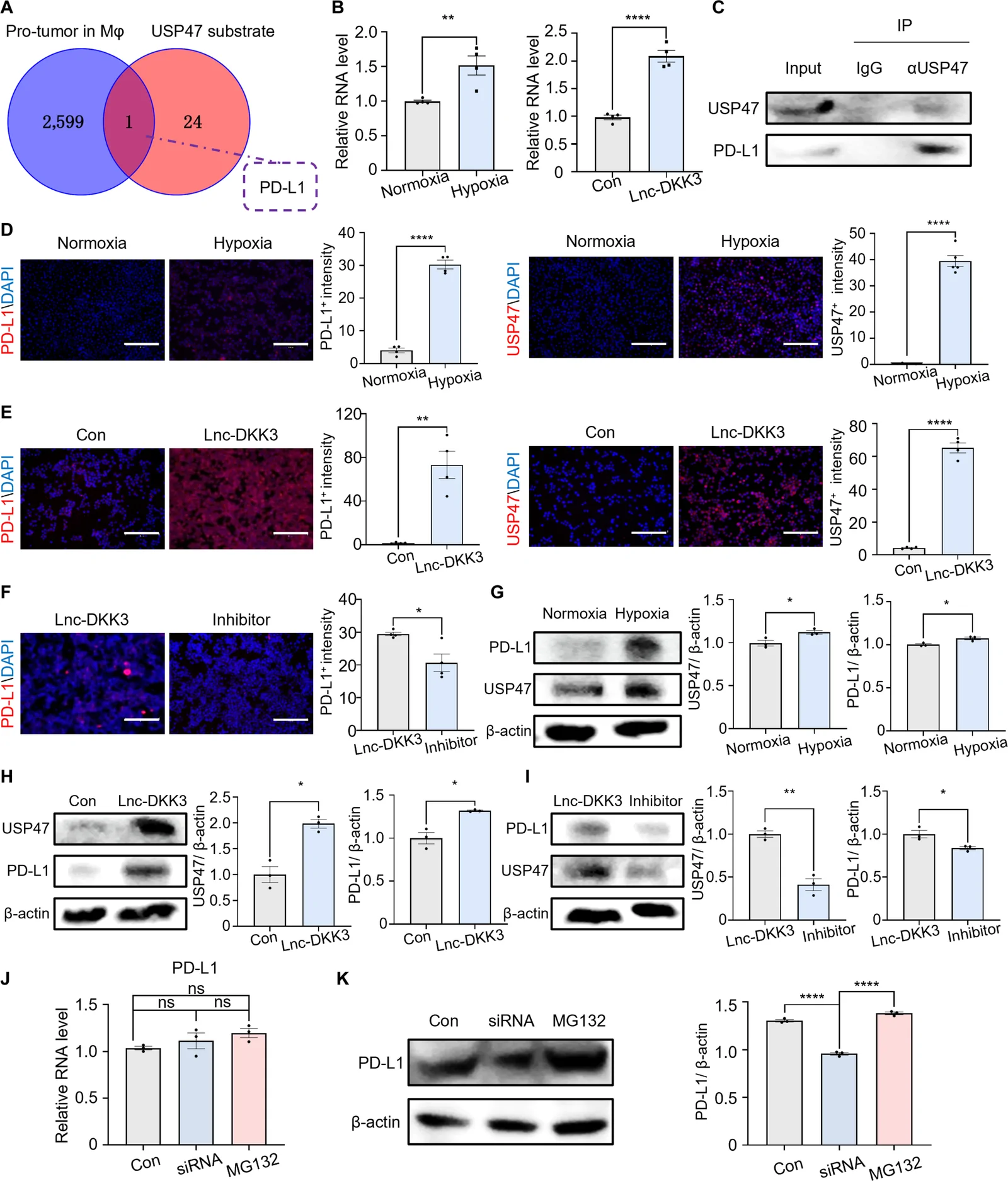
Lnc-DKK3 promotes PD-L1 expression in macrophages by regulating USP47. (A) Bioinformatics analysis revealed a significant association between high PD-L1 expression in pro-tumorigenic macrophages and USP47. (B) qRT-PCR confirmed USP47 up-regulation induced by hypoxic endothelial conditioned medium or lnc-DKK3 overexpression, which was reversed by the USP47 inhibitor. (C) Co-immunoprecipitation (Co-IP) demonstrated a physical interaction between USP47 and PD-L1, which was enhanced upon lnc-DKK3 overexpression. (D to I) Immunofluorescence and Western blot analysis showed increased PD-L1 and USP47 protein levels under hypoxic medium or with lnc-DKK3 overexpression; these levels were reduced by USP47 inhibitor. (J) qRT-PCR showed that USP47 did not affect PD-L1 mRNA levels. (K) MG132 rescue experiments confirmed that USP47 stabilized PD-L1 protein via deubiquitination. The USP47 siRNA sequence was 5′-TGAAAAGGGATGTGCAAAA-3′. *P < 0.05, **P < 0.01, ****P < 0.0001; ns, not significant; 2-tailed unpaired Student *t* test or one-way ANOVA; mean ± SEM; scale bar: 150 μm.

To confirm that exosomal lnc-DKK3 engages the downstream signaling cascade in recipient cells, we isolated exosomes from normoxic and hypoxic HUVEC supernatants and treated iBMDMs with the purified exosomes. Western blot analysis confirmed increased USP47 and PD-L1 following treatment with hypoxic exosomes, demonstrating that exosomal lnc-DKK3 activates the USP47/PD-L1 axis (Fig. S4A to C). Given that USP47 promotes the accumulation of its substrate proteins, we intersected known USP47 deubiquitination substrates with genes involved in pro-tumor immune evasion in TAMs, identifying PD-L1 as a key target whose protein stability is maintained by USP47 (Fig. 6A). Previous studies have shown that USP47 deficiency reduces PD-L1 protein level without affecting its mRNA expression [42]. qRT-PCR analyses confirmed that USP47 mRNA was significantly increased in macrophages treated with hypoxic conditioned medium or overexpressing lnc-DKK3, whereas a USP47 inhibitor suppressed this effect (Fig. 6B). Co-immunoprecipitation assays confirmed a physical interaction between USP47 and PD-L1, which was enhanced upon lnc-DKK3 overexpression (Fig. 6C). Immunofluorescence (Fig. 6D to F) and Western blot analyses (Fig. 6G to I) consistently demonstrated that PD-L1 and USP47 protein expression was up-regulated in macrophages treated with hypoxic conditioned medium or overexpressing lnc-DKK3 and down-regulated by USP47 inhibitor treatment.

To validate the specificity of USP47-mediated effects, we designed 3 independent siRNAs targeting USP47. All 3 siRNAs efficiently knocked down USP47 at both mRNA (Fig. S4D) and protein levels (Fig. S4E). USP47 knockdown significantly attenuated lnc-DKK3 overexpression-induced up-regulation of USP47 at the mRNA level (Fig. S4F) and reversed USP47, PD-L1, and downstream signaling protein up-regulation by Western blot (Fig. S4G). To determine whether USP47 regulates PD-L1 post-translationally, we performed qRT-PCR and found no significant changes in PD-L1 mRNA upon USP47 knockdown or overexpression (Fig. 6J). MG132 rescue experiments further demonstrated that proteasome inhibitor treatment rescued PD-L1 protein levels in USP47-knockdown cells (Fig. 6K), confirming that USP47 stabilizes PD-L1 by protecting it from proteasome-dependent degradation. These results validate the pathway wherein lnc-DKK3 up-regulates PD-L1 expression in macrophages through positive regulation of USP47.

### Lnc-DKK3 up-regulates RelA in glioma cells via USP47 and induces T-cell exhaustion

**Fig. 7.**
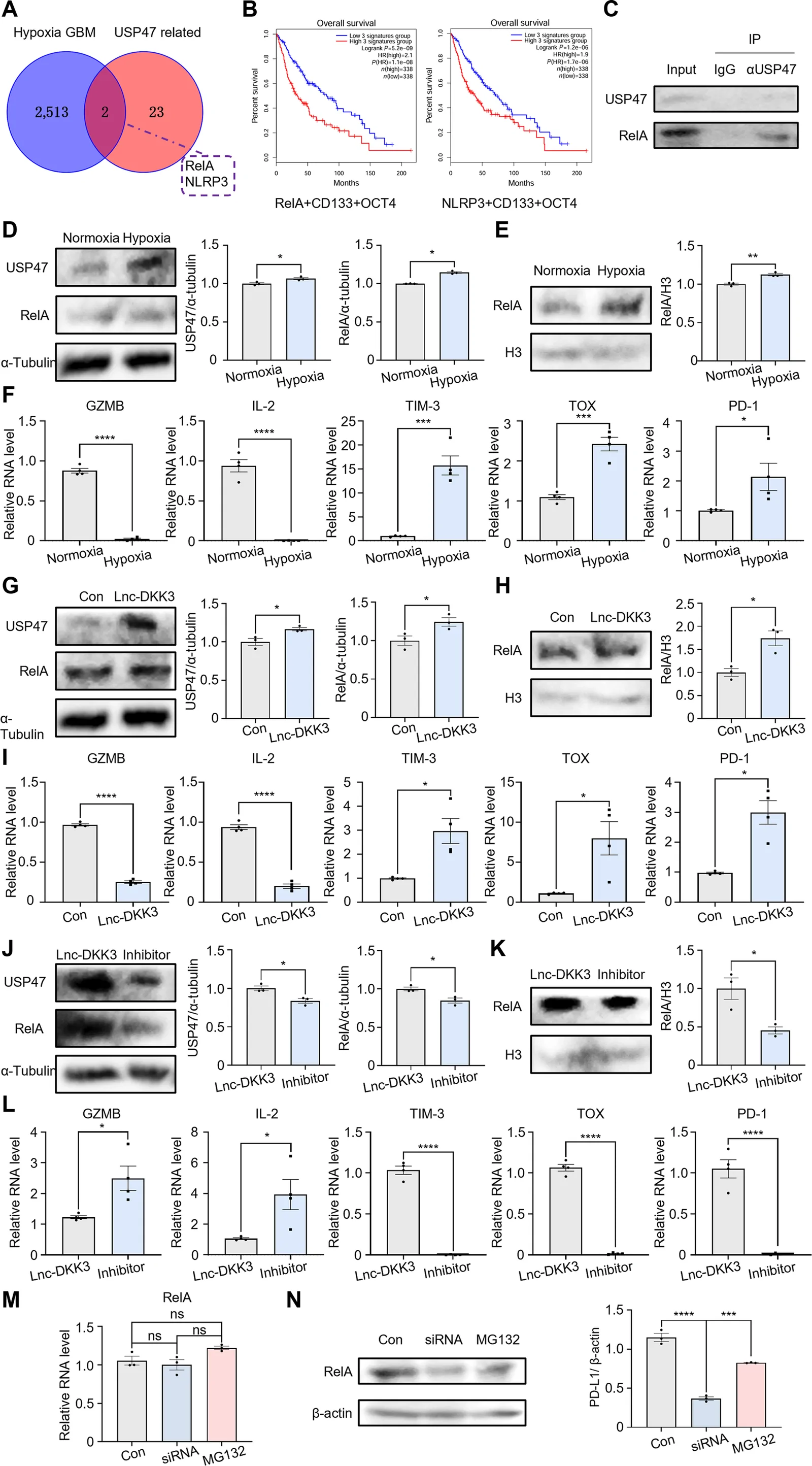
Lnc-DKK3 up-regulates RelA in glioma cells and induces T-cell exhaustion via the USP47/RelA axis. (A and B) Bioinformatics analysis revealed altered gene expression in hypoxic glioma cells and a positive correlation between RelA and USP47. (C) Co-IP confirmed the physical interaction between USP47 and RelA. (D and E) Western blot analysis demonstrated increased RelA nuclear translocation following exposure to hypoxic endothelial conditioned medium. (F) qRT-PCR showed up-regulation of T-cell exhaustion markers in co-cultured T cells. GZMB, Granzyme B; TOX, thymocyte selection-associated high mobility group box protein; IL-2, interleukin-2; TIM-3, T cell immunoglobulin and mucin domain-containing protein 3; PD-1, programmed cell death protein 1. (G to I) Lnc-DKK3 overexpression enhanced RelA nuclear translocation and increased the expression of exhaustion marker expression. (J to L) USP47 inhibition reversed these effects. (M) USP47 did not affect RelA mRNA levels. (N) MG132 rescue experiments confirmed that USP47 stabilizes RelA protein via deubiquitination. *P < 0.05, **P < 0.01, ***P < 0.001, ****P < 0.0001; ns, not significant; 2-tailed unpaired Student *t* test or one-way ANOVA; mean ± SEM.

Exosome-derived lncRNAs play a critical role in tumor progression by promoting malignant phenotypic transformation in glioma and mediating intercellular communication to facilitate tumorigenesis. For instance, the exosomal lncRNA CRNDE-h derived from colorectal cancer promotes Th17 cell differentiation, thereby exerting a pro-tumorigenic effect within the TME [43]. Beyond its established role in gastric cancer, the deubiquitinase USP47 has been shown to sustain NF-κB signaling by stabilizing RelA and promoting chemoresistance [44]. However, the upstream regulatory mechanisms governing USP47 activity within the glioma microenvironment—particularly how paracrine signals from the hypoxic endothelium might influence this axis—remain poorly understood. To confirm that exosomal lnc-DKK3 engages the downstream cascade in glioma cells, exosomes were isolated from normoxic and hypoxic HUVEC supernatants and used to treat GL261 cells. Western blot analysis confirmed increased USP47 and RelA expression following hypoxic exosome treatment, demonstrating activation of the USP47/RelA axis (Fig. S5H and I). To elucidate the role of this axis in glioma malignancy and immune evasion, hypoxia-induced genes were screened against known USP47 deubiquitination substrates; this identified RelA, a key NF-κB subunit, as being associated with the hypoxia-induced phenotype in GBM (Fig. 7A and B). Co-immunoprecipitation assays confirmed a physical interaction between USP47 and RelA in glioma cells (Fig. 7C). Previous studies have demonstrated that USP47 promotes the stability of β-transducin repeat-containing protein and the function of RelA, suggesting that inhibiting USP47 may be a viable strategy to reduce NF-κB activity and overcome chemotherapy resistance [45]. In glioma cells treated with hypoxic endothelial conditioned medium, both RelA and USP47 were up-regulated in the cytoplasmic fraction, accompanied by RelA nuclear translocation and increased T-cell exhaustion markers (Fig. 7D to F), findings that were further confirmed by enzyme-linked immunosorbent assay (ELISA; Fig. S5A). Lnc-DKK3 overexpression recapitulated these effects, including the exacerbation of T-cell exhaustion (Fig. 7G to I and Fig. S5B), while USP47 inhibition reversed them (Fig. 7J to L and Fig. S5C). Cell Counting Kit-8 (CCK8) proliferation assays and flow-cytometry-based cytotoxicity assays confirmed that T cells co-cultured with lnc-DKK3-overexpressing or hypoxic conditioned medium-treated glioma cells exhibited markedly reduced proliferation and killing capacity, which were partially restored by USP47 inhibition (Fig. S5D to G). qRT-PCR revealed no significant changes in RelA mRNA levels upon USP47 knockdown or overexpression (Fig. 7M), and MG132 rescue experiments confirmed that USP47 stabilizes RelA by protecting it from proteasome-dependent degradation (Fig. 7N). These results demonstrate that lnc-DKK3 up-regulates USP47, which stabilizes RelA and promotes its nuclear translocation, ultimately activating RelA-mediated transcription and inducing T-cell exhaustion, thereby driving immune evasion.

### Blocking the lnc-DKK3-bound enhancer of USP47 in M2 macrophages inhibits tumor growth and enhances PD-1 therapy sensitivity in vivo

**Fig. 8.**
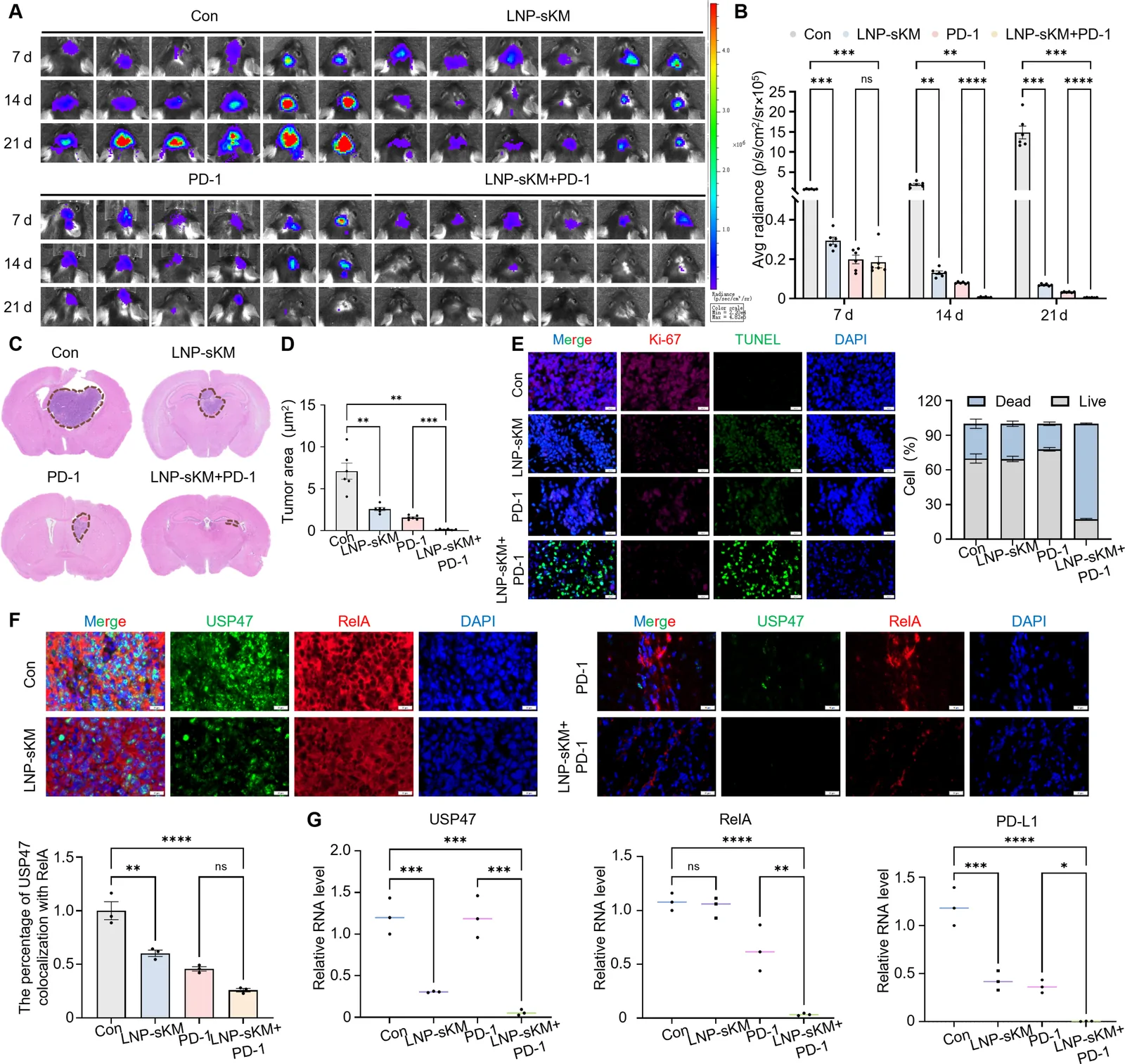
Evaluation of the antitumor effects of targeting the lnc-DKK3-bound enhancer of USP47 in M2 macrophages combined with PD-1 inhibitor. (A and B) In vivo imaging showed significantly reduced intracranial tumor fluorescence in the combination therapy group compared to controls (*n* = 6). (C and D) HE staining confirmed smaller tumor areas in the combination group. (E) Immunofluorescence revealed decreased Ki-67 and increased TUNEL signals in the combination group. (F) USP47 and RelA expression were down-regulated in the combination group. (G) qRT-PCR confirmed reduced mRNA levels of USP47, RelA, and PD-L1 in the combination group. *P < 0.05, **P < 0.01, ***P < 0.001, ****P < 0.0001; ns, not significant; one-way ANOVA; mean ± SEM; scale bar: 50 μm.

**Fig. 9.**
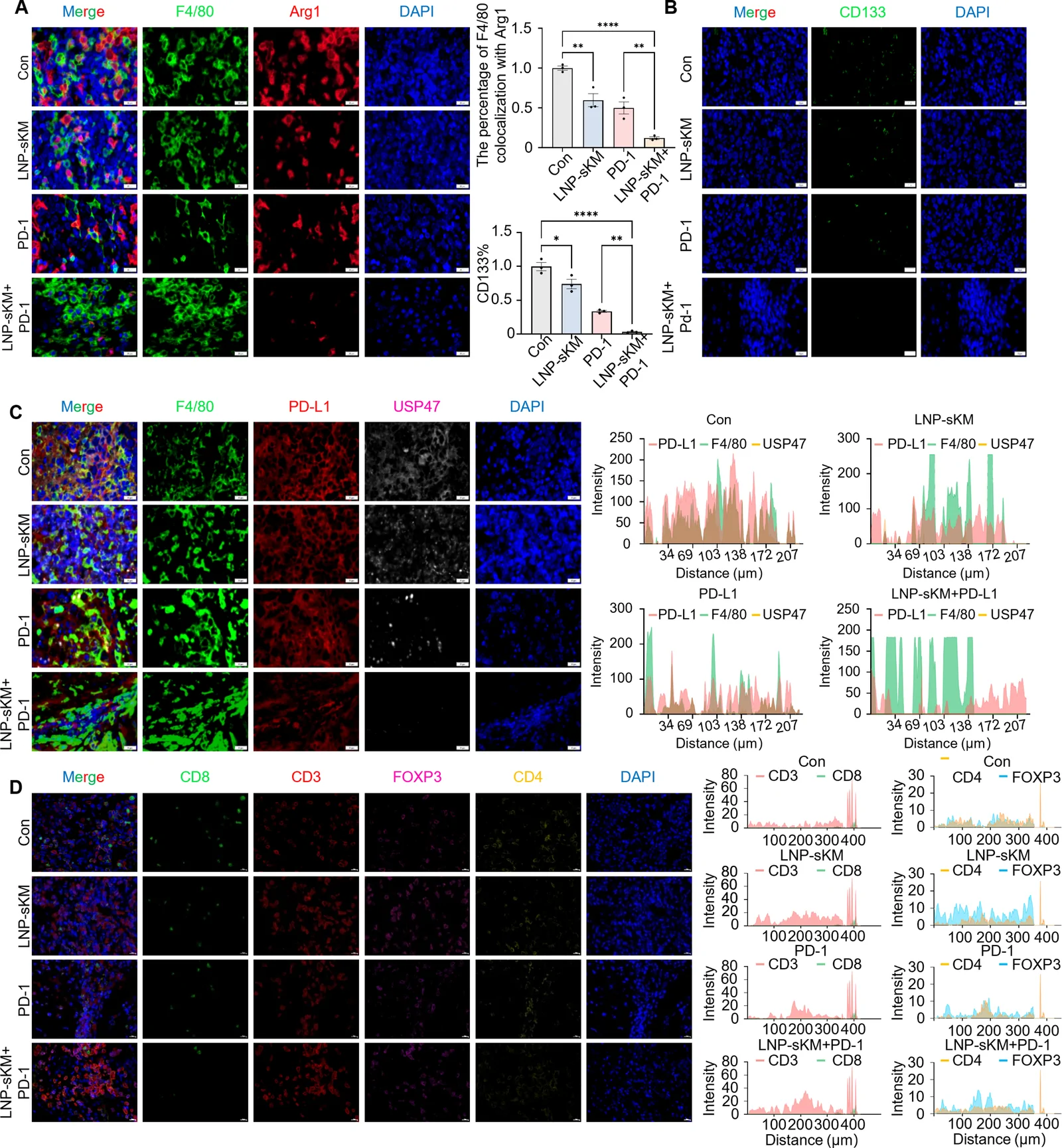
Evaluation of the antitumor effects of targeting the lnc-DKK3-bound enhancer of USP47 in M2 macrophages combined with PD-1 inhibitor in vivo test. (A) Immunofluorescence detection of the proportion of M2 macrophages in tumor tissues. (B) The proportion of glioma stem cells (CD133^+^, green) in tumor tissues was detected by immunofluorescence. (C) Immunofluorescence double staining showing the expression levels and quantification of PD-L1 (red) and USP47 (white) in macrophages (F4/11180^+^, green) from tumor tissues. (D) Multiple immunofluorescence staining showed the expression and statistics of T cells in the immune microenvironment (FOXP3^+^, green; CD4^+^, red; CD8^+^, purple; CD3^+^, yellow). *P < 0.05, **P < 0.01, ****P < 0.0001 one-way ANOVA; mean ± SEM; scale bar: 50 μm.

Based on our systematic investigation of hypoxic endothelial exosomal lnc-DKK3 in remodeling the glioma immune microenvironment, we employed a CRISPR interference (CRISPRi) gene editing system to construct [46,47] lnc-DKK3 sgRNA@KRAB@M2pep@LNP(LNP-sKM), which specifically targets and suppresses the lnc-DKK3-bound enhancer of USP47 specifically in M2 macrophages. LNP-sKM demonstrated favorable safety profiles and specific uptake by M2-polarized macrophages (Fig. S6A and B). In a mouse glioma model, in vivo imaging revealed that LNP-sKM combined with anti-PD-1 therapy exerted the most potent tumor-suppressive effect (Fig. 8A and B). Survival analysis confirmed significantly prolonged median survival in the combination group, with 30% of mice surviving beyond 50 d (Fig. S6C). Body-weight monitoring showed that combination-treated mice gradually gained weight, whereas controls lost weight due to tumor burden (Fig. S6D). Furthermore, the combination treatment resulted in Ki-67 down-regulation and terminal deoxynucleotidyl transferase dUTP nick end labeling (TUNEL) up-regulation (Fig. 8E). Mechanistically, combination therapy decreased USP47, RelA, and PD-L1 expression at both protein and mRNA levels (Fig. 8F and G). Western blot analysis of brain tumor tissues revealed that LNP-sKM treatment significantly reduced USP47 protein levels, confirming effective target silencing in vivo (Fig. S6E). ELISA of tumor tissue lysates demonstrated that combination therapy significantly increased the levels of pro-inflammatory cytokines (IL-1β, TNF-α, and IFN-γ) and the T-cell chemoattractant CXCL10, indicating a shift toward an antitumor immune microenvironment (Fig. S6F). Consistent with these findings, qRT-PCR analysis showed reduced expression of the M2 marker Arg1 and elevated expression of pro-inflammatory cytokines (IL-1β, TNF-α, and IFN-γ) in the combination therapy group (Fig. S6G). Additionally, combination therapy also reduced M2 macrophage markers (Fig. 9A), decreased CD133^+^ glioma stem cells (Fig. 9B), suppressed USP47 and PD-L1 expression in macrophages (Fig. 9C), and remodeled the immune microenvironment by reducing FOXP3^+^ Tregs, increasing CD8^+^ T cells and alleviating T-cell exhaustion (Fig. 9D). These results demonstrate that targeting the lnc-DKK3-bound enhancer in M2 macrophages remodels the immune microenvironment and enhances anti-PD-1 efficacy, providing a novel combination immunotherapy strategy for glioma.

## Discussion

**Fig. 10.**
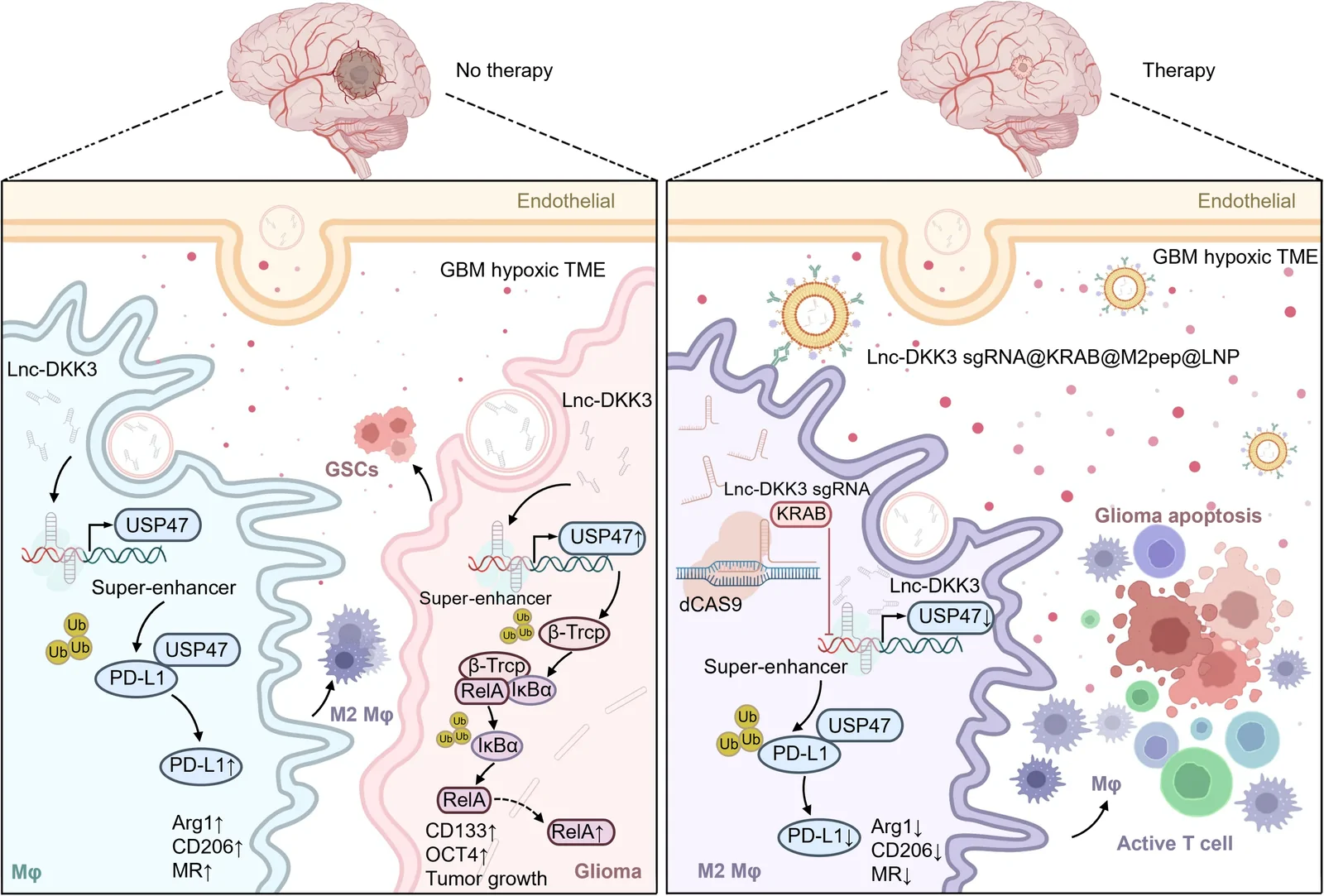
Schematic diagram of lncRNA lnc-DKK3 from hypoxic endothelial-cell-derived exosomes reprogramming tumor-associated macrophages through the USP47/PD-L1/RelA axis to promote glioma progression. TME, tumor microenvironment; GSC, glioma stem cell.

This study proposes a promising combinatorial immunotherapy strategy for glioma. To investigate the impact of the hypoxic microenvironment on endothelial paracrine signaling, we successfully blocked endothelial exosome secretion, which significantly inhibited glioma progression and remodeled the immune microenvironment. Compositional analysis of hypoxic endothelial-cell-derived exosomes revealed altered expression of several nucleus-localized lncRNAs, with lnc-DKK3 being notably up-regulated. Through multidimensional validation, we demonstrated that lnc-DKK3 directly binds to and *cis*-activates the USP47 enhancer: CRISPRi-mediated silencing of the enhancer reduced USP47 expression, dual-luciferase reporter assays confirmed *trans*-activation, and ChIP-qPCR assays revealed direct physical interaction with the USP47 enhancer chromatin. Bioinformatic analysis indicated that the lnc-DKK3 locus contains an enhancer capable of binding to and promoting USP47 transcription. Mechanistically, lnc-DKK3 delivered to macrophages up-regulates USP47 to stabilize PD-L1, driving M2-like polarization; concurrently, in glioma cells, the lnc-DKK3/USP47 axis promotes RelA nuclear translocation, up-regulates PD-L1, and induces T-cell exhaustion. Finally, we constructed a CRISPRi system targeting the lnc-DKK3 super-enhancer, which, in combination with PD-L1 blockade, activated antitumor immunity in macrophages and suppressed stemness, demonstrating marked synergistic efficacy (Fig. 10).

The complexity of the glioma microenvironment is not limited to immunosuppression. Nonimmune elements, particularly endothelial cells as key drivers, are critical contributors to tumor progression and therapeutic resistance [48]. The vascular endothelium drives tumor progression through a dual mechanism: structurally, the imbalance between angiogenesis and vascular normalization supports growth and facilitates metastasis [49,50]; functionally, its dynamic paracrine signaling actively remodels microenvironmental homeostasis [8,51]. The pervasive hypoxia in glioma profoundly alters the endothelial secretory profile, especially exosomal cargo, which in turn modulates macrophages, immunosuppression, cellular functions, and stem-cell proliferation [52], rendering the stem-cell niche a key target for directed therapy and sensitization to resistance [53]. Accordingly, we investigated how hypoxia-mediated endothelial paracrine signals affect stem-cell niche architecture and maintenance. We found that blocking hypoxic endothelial exosomes significantly inhibited tumor progression, reshaped the immune microenvironment, and suppressed stem-cell proliferation, suggesting that targeting functional factors within these exosomes holds therapeutic promise. However, direct inhibition of endothelial exosome components has shown suboptimal efficacy [54] and faces clinical challenges such as poor targeting and low specificity [55], underscoring the need for deeper mechanistic insight. We identified nucleus-localized lnc-DKK3 in hypoxic endothelial exosomes as a pivotal regulator of glioma immune modulation. Unlike prior research focused on tumor-derived exosomal transcripts, this finding provides a novel perspective on the hypoxia–endothelium–tumor immune axis and bridges a critical gap in glioma paracrine immune signaling.

Previous studies indicate that direct endothelial cell–tumor interactions within the hypoxic microenvironment can modulate exosomal secretion from both cell types [56]. As key intercellular messengers, exosomes transport proteins, lipids, and various RNAs [9]. The functional significance of exosomal cargo has been debated due to its minute quantities—with some arguing that membrane physical properties or protein content dominate the observed effects [8]. Our study, however, provides evidence for the functional activity of RNA cargo, raising a fundamental question: How do trace quantities of RNA drive profound biological effects? Conflicting data suggest that this powerful regulatory capacity relies on intrinsic molecular mechanisms that support signal amplification in recipient cells [57,58]. Recently, a class of nucleus-localized lncRNAs has drawn attention for the ability to bind enhancers at very low copy numbers and *trans*-regulate numerous downstream genes. For instance, LINC01116 promotes astrocytic malignant transformation [59], LINC02774 is negatively correlated with glioma grade [60], and HIF1α-AS1 regulates endothelial function through triplex structures [61]. In this study, we identified lnc-DKK3 as a markedly enriched nucleus-localized lncRNA in hypoxic endothelial exosomes. Furthermore, we detected a super-enhancer (SE) at its locus, and Hi-C data indicated direct interaction with the USP47 promoter. SEs, which are clusters of multiple enhancers, represent essential epigenetic oncogenic factors critical for maintaining cancer cell identity [62]. In glioma, lncRNA-mediated long-range enhancer–promoter interactions (e.g., HOXD-AS2 and LINC01116) regulate tumor survival [63], dynamic enhancer RNA expression modulates recurrence-associated transcription [64,65], and Cx43 influences resistance via the T-cell factor/lymphoid enhancer factor pathway [66]. Building upon this framework, we demonstrate that lnc-DKK3 delivered to macrophages up-regulates USP47 to drive M2 polarization and directly acts on glioma cells to enhance proliferation, stemness, and invasion. We further elucidated the mechanisms by which hypoxia drives endothelial secretion of lnc-DKK3-enriched exosomes to establish a paracrine immunosuppressive loop; how this axis simultaneously promotes M2 polarization and via PD-L1 and maintains stemness via RelA; and how it integrates hypoxia sensing, exosomal communication, and posttranslational modification as a nodal point in the TME. These findings suggest that targeting the lnc-DKK3–SE interaction represents a key therapeutic strategy for treating glioma and overcoming immunotherapy resistance, and that the lnc-DKK3/USP47 co-expression signature holds potential as a composite prognostic biomarker.

Conventional methods for targeted knockdown or inhibition of lncRNAs, including ASO/GapmeR, RNA interference, and CRISPR-Cas genome editing technologies, exhibit substantial limitations [67,68]. Given that most lncRNAs function in concert with coding genes, and direct targeting often incurs off-target effects [69], we propose that designing sequences to target the specific interaction between the lnc-DKK3 SE and the USP47 promoter may circumvent nonspecific regulation. Our prior research in liver cancer showed that deleting specific enhancers or CpG islands could ameliorate cancer progression [70]. However, clinical feasibility remains limited, rendering the suppression of SE activation a pivotal strategy. Consequently, we therefore employed CRISPRi with a dCas9-KRAB fusion protein to specifically silence the USP47 enhancer bound by lnc-DKK3, a mechanism operating through steric hindrance and the recruitment of KAP1/HP1α to establish a repressive chromatin environment for long-term silencing [71,72]. In a murine glioma model, LNP-delivered CRISPRi effectively inhibited tumor growth and synergized with PD-1 blockade. Notably, this study utilized an immunocompetent GL261 syngeneic mouse glioma model; while widely used and amenable to mechanistic dissection, this model does not fully recapitulate the high heterogeneity and complex pathological features of human GBM. Therefore, the translational value of this combination therapy necessitates further validation in more clinically relevant models, such as patient-derived xenografts or genetically engineered mouse models. We acknowledge that the 21-d endpoint was selected to prioritize mechanistic analysis, and long-term survival studies are warranted. The translational potential of this approach also depends on the efficiency of LNP delivery to human TAMs and the feasibility of clinical-grade CRISPRi manufacturing, both of which remain to be addressed. Furthermore, macrophage-specific targeting relies on the M2pep ligand, whose specificity within the complex human TME—characterized by continuous macrophage phenotypic plasticity—requires rigorous validation. Despite these limitations, this LNP-delivered CRISPRi platform targeting disease-specific SEs represents a promising innovative therapeutic paradigm for overcoming immunotherapy resistance in glioma.

## Materials and Methods

### Cell culture and hypoxic treatment

GL261 glioma cells and iBMDMs were maintained in high-glucose Dulbecco’s Modified Eagle Medium with 10% fetal bovine serum (FBS) and 1% antibiotics; HUVECs were cultured in endothelial medium with 5% FBS, endothelial cell growth supplement, and antibiotics, all at 37 °C and 5% CO_2_. Hypoxia was induced by incubating cells in 1% O_2_, 5% CO_2_, and 94% N_2_ for 24 to 72 h, with normoxia (21% O_2_) as control. Conditioned medium from HUVECs cultured under normoxia or hypoxia for 48 h was collected, filtered (0.22 μm), and used fresh or stored at –80 °C.

### Detailed bioinformatic workflow including databases used, analysis parameters, and statistical methods

RNA-sequencing raw reads were processed with fastp for quality filtering, Bowtie2 for rRNA removal, and HISAT2 for alignment. Transcripts were assembled by StringTie, and values of fragments per kilobase of transcript per million mapped reads were calculated; novel transcripts and lncRNAs were identified using Cuffcompare, Coding-Non-Coding Index, and Coding Potential Calculator. Sample consistency was assessed by Pearson correlation and principal component analysis. Differentially expressed genes were identified with DESeq2/edgeR (false discovery rate ≤ 0.05). Additional analyses included miRNA prediction, untranslated-region optimization, variant filtering, splicing detection, and antisense pairing prediction; *cis* targets were defined within ±100 kb and *trans* targets via correlation or WGCNA.

### Exosome isolation and characterization protocol

Exosomes were isolated from cell culture supernatants and urine by sequential centrifugation (10,000×*g*, 30 min), concentration via 100-kDa ultrafiltration (3,500×*g*, 15 min), filtration (0.22 μm), and ultracentrifugation (120,000×*g*, 90 min). Nanoparticle tracking analysis (ZetaView) revealed particle concentrations of 1.8 × 10^8^ particles/ml (normoxia, median 97.1 nm) and 1.7 × 10^8^ particles/ml (hypoxia, median 100.6 nm). Transmission electron microscopy showed typical cup-shaped morphology. Protein concentrations were determined using the bicinchoninic acid assay, and equal amounts of total protein (20 μg per lane) were loaded for sodium dodecyl sulfate–polyacrylamide gel electrophoresis, followed by transfer to polyvinylidene fluoride membranes. Western blot confirmed positivity for CD9, CD81, and TSG101 and negativity for Calnexin. NanoFCM detected surface marker positive rates.

### In vitro functional assays.

Cell viability was measured by Cell Counting Kit-8. Clonogenic ability was assessed by seeding 1,000 cells/well for 10 to 14 d, staining colonies (>50 cells) with crystal violet. Migration was evaluated by wound healing assay (scratch, images at 0, 12, and 24 h) with ImageJ quantification. Macrophage polarization was induced in iBMDMs with IFN-γ (20 ng/ml) + lipopolysaccharide (50 ng/ml) for M1, or IL-4 (20 ng/mL) for M2, for 24 h; alternatively, lnc-DKK3 overexpression was used. Polarization was assessed by qRT-PCR and flow cytometry for M1 (iNOS and CD86) and M2 (Arg1 and CD206) markers.

### Molecular analyses

Total RNA was extracted with TRIzol and reverse transcribed, and qRT-PCR was performed with SYBR Green to measure lnc-DKK3, USP47, M2 markers, stemness genes, and T-cell exhaustion markers, normalized to β-actin. RNA expression was calculated as the fold change in the expression of each target gene relative to the control sample using the 2^−ΔΔCt^ method. Proteins were extracted with radioimmunoprecipitation assay, quantified by bicinchoninic acid assay, resolved by sodium dodecyl sulfate–polyacrylamide gel electrophoresis, transferred to polyvinylidene fluoride membranes, and immunoblotted with antibodies against USP47, PD-L1, RelA, α-tubulin, H3, etc., followed by horseradish peroxidase-secondary and enhanced chemiluminescence detection. Immunofluorescence was performed on cells/tissue sections with primary antibodies overnight at 4 °C, then Alexa Fluor-conjugated secondaries and DAPI, imaged by confocal microscopy.

### T-cell isolation and co-culture of GL261 glioma cells and T cells

Primary CD3^+^ T cells were isolated from spleens of 8- to 12-week-old C57BL/6 mice using magnetic bead negative selection, activated with anti-CD3/CD28 beads, and co-cultured with GL261 target cells at a 10:1 ratio in RPMI 1640 complete medium (with 10% FBS, β-mercaptoethanol, sodium pyruvate, and L-glutamine) for 24 h. Before co-culture, GL261 cells were preconditioned for 24 h with hypoxic HUVEC-conditioned medium (1:1 with Dulbecco’s Modified Eagle Medium) or 10 μM USP47 inhibitor, then washed twice. After co-culture, suspended T cells were harvested for qRT-PCR of exhaustion markers, and supernatants for ELISA.

### Dual-luciferase reporter assay

The USP47 SE fragment was cloned to drive firefly luciferase; the full lnc-DKK3 transcript was cloned for overexpression; pRL-TK served as internal control. Cells at 70% to 80% confluence were transfected, and after 48 h, firefly and Renilla activities were measured sequentially. Relative luciferase activity was calculated as firefly/Renilla ratio, with ≥3 replicates, and analysis of variance (ANOVA) followed by Tukey test.

### CRISPRi system

The CRISPRi system was constructed with a dual-sgRNA cassette targeting the mouse lnc-DKK3 locus (LOC132412331), using sgRNA sequences GTTAACTACAGATCAAGCAG and TAAAGACTAGCACAAAGAAC, cloned into the GV469 backbone at MluI/HindIII sites, with the non-targeting control. The dCas9-KRAB effector was generated in the GV712 backbone via XbaI cloning, producing a 4,508-bp insert encoding the full-length dCas9–KRAB fusion protein.

### Synthesis of LNPs functionalized with various targeting ligands

APRPG@Nexinhib20@LNP was prepared by dissolving DLin-MC3-DMA, DSPC, cholesterol, DSPE-PEG2000-APRPG, and Nexinhib20 in ethanol (40:10:35:10:5 molar ratio), rapidly injecting into citrate buffer (pH 4), equilibrating, and extruding through 80-nm membranes to obtain 70- to 90-nm vesicles. KRAB@M2pep@LNP (for CRISPRi in M2 macrophages) was formulated using preformed vesicles of DLin-MC3-DMA, DSPC, cholesterol, and DSPE-PEG2000-M2pep (40:10:40:10), then mixed with an equimolar mixture of dCas9-KRAB and sgRNA plasmids in citrate buffer/ethanol, incubated at 35 °C for 30 min, and purified by ultrafiltration. Both were stored at 4 °C.

### Statistical analysis

Gene correlations and survival analyses were performed with GEPIA2 using TCGA glioma data and with median cutoff and log-rank test. All in vitro experiments had ≥3 replicates; data were presented as mean ± SD; 2-group comparisons were performed using an unpaired *t* test, multigroup by one-way ANOVA; *P* <0.05 was considered significant.

### In vivo therapeutic experiments.

For in vivo studies, male C57BL/6 mice (8 to 12 wk) were housed under specific pathogen-free conditions. Orthotopic glioma was established by stereotactic injection of luciferase-expressing GL261 cells (1 × 10^5^ in 2 μl phosphate-buffered saline) into the right striatum. Tumor growth was monitored by bioluminescence imaging on days 7, 14, and 21; mice were randomized by signal intensity. At endpoint, transcardial perfusion was performed, brains fixed or snap-frozen, and tumor area/fluorescence quantified with Image-Pro Plus. All animal procedures were approved by the institutional committee.

## Data Availability

All data supporting the findings of this study are provided within the paper and its supplementary materials. Sequence data included in this study have been deposited in the Gene Expression Omnibus of the National Genomics Data Center, under accession number OMIX019012.

## References

[1] Greenwald AC, Darnell NG, Hoefflin R, Simkin D, Mount CW, Castro LN, Harnik Y, Dumont S, Hirsch D, Nomura M, et al. Integrative spatial analysis reveals a multi-layered organization of glioblastoma. Cell. 2024;187(10):2485–2501. 38653236 10.1016/j.cell.2024.03.029 PMC11088502

[2] Wang W, Li T, Cheng Y, Li F, Qi S, Mao M, Wu J, Liu Q, Zhang X, Li X, et al. Identification of hypoxic macrophages in glioblastoma with therapeutic potential for vasculature normalization. Cancer Cell. 2024;42(5):815–832. 38640932 10.1016/j.ccell.2024.03.013

[3] Shin S, Choi Y, Jang W, Ulziituya B, Ha G, Kang R, Park S, Kim M, Zhang YS, Kim HJ, et al. A vascularized tumors-on-a-chip model for studying tumor-angiogenesis interplay, heterogeneity and drug responses. Mater Today Bio. 2025;32: Article 101741. 10.1016/j.mtbio.2025.101741 PMC12020855 40275956

[4] Kim D, Hwang KS, Seo EU, Seo S, Lee BC, Choi N, Choi J, Kim HN. Vascularized lung cancer model for evaluating the promoted transport of anticancer drugs and immune cells in an engineered tumor microenvironment. Adv Healthc Mater. 2022;11(12): Article e2102581. 35286780 10.1002/adhm.202102581 PMC11468795

[5] Greenspan LJ, Weinstein BM. To be or not to be: Endothelial cell plasticity in development, repair, and disease. Angiogenesis. 2021;24(2):251–269. 33449300 10.1007/s10456-020-09761-7 PMC8205957

[6] Zhang L, Xu J, Zhou S, Yao F, Zhang R, You W, Dai J, Yu K, Zhang Y, Baheti T, et al. Endothelial DGKG promotes tumor angiogenesis and immune evasion in hepatocellular carcinoma. J Hepatol. 2024;80(1):82–98. 37838036 10.1016/j.jhep.2023.10.006

[7] Peng H, Zhu E, Zhang Y. Advances of cancer-associated fibroblasts in liver cancer. Biomark Res. 2022;10(1):59. 35971182 10.1186/s40364-022-00406-z PMC9380339

[8] Mu Y, Yang M, Liu J, Yao Y, Sun H, Zhuang J. Exosomes in hypoxia: Generation, secretion, and physiological roles in cancer progression. Front Immunol. 2025;16:1537313. 40534881 10.3389/fimmu.2025.1537313 PMC12174069

[9] Guo W, Qiao T, Dong B, Li T, Liu Q, Xu X. The effect of hypoxia-induced exosomes on anti-tumor immunity and its implication for immunotherapy. Front Immunol. 2022;13: Article 915985. 35812406 10.3389/fimmu.2022.915985 PMC9257077

[10] Wang C, Xu S, Yang X. Hypoxia-driven changes in tumor microenvironment: Insights into exosome-mediated cell interactions. Int J Nanomedicine. 2024;19:8211–8236. 39157736 10.2147/IJN.S479533 PMC11328847

[11] Liu C, Luo Y, Zhou H, Lin M, Zang D, Chen J. Immune cell-derived exosomal non-coding RNAs in tumor microenvironment: Biological functions and potential clinical applications. Chin J Cancer Res. 2025;37(2):250–267. 40353080 10.21147/j.issn.1000-9604.2025.02.10 PMC12062983

[12] Tang T, Yang T, Xue H, Liu X, Yu J, Liang C, Li D, Xiang C, Zheng J, Wei L, et al. Breast cancer stem cell-derived exosomal lnc-PDGFD induces fibroblast-niche formation and promotes lung metastasis. Oncogene. 2025;44(9):601–617. 39633064 10.1038/s41388-024-03237-4 PMC11850284

[13] Wang D, Zhang W, Zhang C, Wang L, Chen H, Xu J. Exosomal non-coding RNAs have a significant effect on tumor metastasis. Mol Ther Nucleic Acids. 2022;29:16–35. 35784014 10.1016/j.omtn.2022.05.034 PMC9207556

[14] Qin Z, Bian X, Shi Y. Identification of hypoxic macrophages in glioblastoma: Unveiling therapeutic insights from tumour microenvironment analysis. Clin Transl Med. 2024;14(9): Article e70013. 10.1002/ctm2.70013 PMC11412069 39297872

[15] Cai S, Chen Z, Wang Y, Wang M, Wu J, Tong Y, Chen L, Lu C, Yang H. Reducing PD-L1 expression with a self-assembled nanodrug: An alternative to PD-L1 antibody for enhanced chemo-immunotherapy. Theranostics. 2021;11(4):1970–1981. 33408792 10.7150/thno.45777 PMC7778587

[16] Rana R, Devi SN, Bhardwaj AK, Yashavarddhan MH, Bohra D, Ganguly NK. Exosomes as nature’s nano carriers: Promising drug delivery tools and targeted therapy for glioma. Biomed Pharmacother. 2025;182: Article 117754. 39731936 10.1016/j.biopha.2024.117754

[17] Inocencio JF, Mitrasinovic S, Asad M, Parney IF, Zang X, Himes BT. Immune checkpoint pathways in glioblastoma: A diverse and evolving landscape. Front Immunol. 2024;15:1424396. 39346924 10.3389/fimmu.2024.1424396 PMC11427296

[18] Liu T, Sun T, Chen X, Wu J, Sun X, Liu X, Yan H, Fu Q, Fan Z, Wang X, et al. Targeting ARPC1B overcomes immune checkpoint inhibitor resistance in glioblastoma by reversing protumorigenic macrophage polarization. Cancer Res. 2025;85(7):1236–1252. 39841088 10.1158/0008-5472.CAN-24-2286

[19] Musatova O, Kumar V, Vinogradov K, Rubtsov Y. Immune checkpoints in immune response to glioma: Two sides of the same coin. Front Immunol. 2025;16:1639521. 40895574 10.3389/fimmu.2025.1639521 PMC12394979

[20] Zhang S, Zhang L, Guan N, Feng X, Lu M, Wang Y. Unveiling the role of TAM-derived extracellular vesicles in glioma progression through Treg polarization and immune suppression. Oncogene. 2025;44(36):3364–3385. 40681801 10.1038/s41388-025-03497-8

[21] Lin YJ, Wu CY, Wu JY, Lim M. The role of myeloid cells in GBM immunosuppression. Front Immunol. 2022;13: Article 887781. 35711434 10.3389/fimmu.2022.887781 PMC9192945

[22] Xu X, Zheng Y, Luo L, You Z, Chen H, Wang J, Zhang F, Liu Y, Ke Y. Glioblastoma stem cells deliver ABCB4 transcribed by ATF3 via exosomes conferring glioblastoma resistance to temozolomide. Cell Death Dis. 2024;15(5):318. 38710703 10.1038/s41419-024-06695-6 PMC11074105

[23] Bayik D, Bartels CF, Lovrenert K, Watson DC, Zhang D, Kay K, Lee J, Lauko A, Johnson S, Lo A, et al. Distinct cell adhesion signature defines glioblastoma myeloid-derived suppressor cell subsets. Cancer Res. 2022;82(22):4274–4287. 36126163 10.1158/0008-5472.CAN-21-3840 PMC9664137

[24] Adnani L, Kassouf J, Meehan B, Spinelli C, Tawil N, Nakano I, Rak J. Angiocrine extracellular vesicles impose mesenchymal reprogramming upon proneural glioma stem cells. Nat Commun. 2022;13(1):5494. 36123372 10.1038/s41467-022-33235-7 PMC9485157

[25] Muthukrishnan SD, Kawaguchi R, Nair P, Prasad R, Qin Y, Johnson M, Wang Q, VanderVeer-Harris N, Pham A, Alvarado AG, et al. P300 promotes tumor recurrence by regulating radiation-induced conversion of glioma stem cells to vascular-like cells. Nat Commun. 2022;13(1):6202. 36261421 10.1038/s41467-022-33943-0 PMC9582000

[26] Li Y, Chen ZK, Duan X, Zhang HJ, Xiao BL, Wang KM, Chen G. Targeted inhibition of tumor-derived exosomes as a novel therapeutic option for cancer. Exp Mol Med. 2022;54(9):1379–1389. 36117219 10.1038/s12276-022-00856-3 PMC9534887

[27] Lerra L, Panatta M, Bär D, Zanini I, Tan JY, Pisano A, Mungo C, Baroux C, Panse VG, Marques AC, et al. An RNA-dependent and phase-separated active subnuclear compartment safeguards repressive chromatin domains. Mol Cell. 2024;84(9):1667–1683. 38599210 10.1016/j.molcel.2024.03.015 PMC11065421

[28] Khan M, Hou S, Chen M, Lei H. Mechanisms of RNA export and nuclear retention. Wiley Interdiscip Rev. 2023;14(3): Article e1755. 10.1002/wrna.1755 35978483

[29] Wang J, Huang X, Zheng D, Li Q, Mei M, Bao S. PRMT5 determines the pattern of polyploidization and prevents liver from cirrhosis and carcinogenesis. J Genet Genom. 2023;50(2):87–98. 10.1016/j.jgg.2022.04.008 35500745

[30] Xu Y, Ren W, Li Q, Duan C, Lin X, Bi Z, You K, Hu Q, Xie N, Yu Y, et al. LncRNA Uc003xsl.1-mediated activation of the NFκB/IL8 axis promotes progression of triple-negative breast cancer. Cancer Res. 2022;82(4):556–570. 34965935 10.1158/0008-5472.CAN-21-1446 PMC9359739

[31] Zhang M, Wu J, Zhong W, Zhao Z, He W. DNA-methylation-induced silencing of DIO3OS drives non-small cell lung cancer progression via activating hnRNPK-MYC-CDC25A axis. Mol Ther Oncol. 2021;23:205–219. 10.1016/j.omto.2021.09.006 PMC8551476 34761103

[32] Jia M, Shen X, Tang Y, Shi X, Gu Y. A karyopherin constrains nuclear activity of the NLR protein SNC1 and is essential to prevent autoimmunity in Arabidopsis. Mol Plant. 2021;14(10):1733–1744. 34153500 10.1016/j.molp.2021.06.011

[33] Chen G, Xu D, Liu Q, Yue Z, Dai B, Pan S, Chen Y, Feng X, Hu H. Regulation of FLC nuclear import by coordinated action of the NUP62-subcomplex and importin β SAD2. J Integr Plant Biol. 2023;65(9):2086–2106. 37278318 10.1111/jipb.13540

[34] Liu S, Kang M, Ren Y, Zhang Y, Ba Y, Deng J, Luo P, Cheng Q, Xu H, Weng S, et al. The interaction between vasculogenic mimicry and the immune system: Mechanistic insights and dual exploration in cancer therapy. Cell Prolif. 2025;58(6): Article e13814. 39865437 10.1111/cpr.13814 PMC12179562

[35] Feichtenschlager V, Chen L, Zheng YJ, Ho W, Sanlorenzo M, Vujic I, Fewings E, Lee A, Chen C, Callanan C, et al. The therapeutically actionable long non-coding RNA ‘T-RECS’ is essential to cancer cells’ survival in NRAS/MAPK-driven melanoma. Mol Cancer. 2024;23(1):40. 38383439 10.1186/s12943-024-01955-7 PMC10882889

[36] Yan Y, Luo A, Liu S, Cai M, Liu X, Zhang X, Zhang S, Liu Y, Zeng J, Xu X, et al. METTL3-mediated LINC00475 alternative splicing promotes glioma progression by inducing mitochondrial fission. Research. 2024;7:324. 10.34133/research.0324 PMC10886067 38405130

[37] Zhang Y, Hudson K, Abounader R. Unraveling the hypoxic puzzle: LncRNA LUCAT1 drives glioblastoma in cooperation with HIF1α. Neuro-Oncology. 2024;26(8):1402–1404. 38769725 10.1093/neuonc/noae096 PMC11299999

[38] Cai XJ, Fei WD, Xu YY, Xu H, Yang GY, Cao JW, Ni JJ, Tao K, Wang Z. Liposome-encapsulated zoledronate favors tumor vascular normalization and enhances anticancer efficacy of cisplatin. AAPS PharmSciTech. 2020;21(2):57. 31912318 10.1208/s12249-019-1614-6

[39] Asai T, Oku N. Angiogenic vessel-targeting DDS by liposomalized oligopeptides. *Methods Mol Biol* 2010;605:335–347. 10.1007/978-1-60327-360-2_23 20072892

[40] Irep N, Inci K, Tokgun PE, Tokgun O. Exosome inhibition improves response to first-line therapy in small cell lung cancer. J Cell Mol Med. 2024;28(4): Article e18138. 38353469 10.1111/jcmm.18138 PMC10865916

[41] Silvestrini VC, Thome CH, Albuquerque D, de Souza Palma C, Ferreira GA, Lanfredi GP, Masson AP, Delsin LE, Ferreira FU, de Souza FC, et al. Proteomics analysis reveals the role of ubiquitin specific protease (USP47) in epithelial to mesenchymal transition (EMT) induced by TGFβ2 in breast cells. J Proteome. 2020;219: Article 103734. 10.1016/j.jprot.2020.103734 32201364

[42] Jiang Y, Yu Z, Wang J, Wu Y, Li Z, Le T, Yang C, Wei Y, Zhang G, Ma H. Targeting USP47 enhances immunotherapy in hepatocellular carcinoma by destabilizing PD-L1. Int Immunopharmacol. 2025;161: Article 115024. 40494207 10.1016/j.intimp.2025.115024

[43] Sun J, Jia H, Bao X, Wu Y, Zhu T, Li R, Zhao H. Tumor exosome promotes Th17 cell differentiation by transmitting the lncRNA CRNDE-h in colorectal cancer. Cell Death Dis. 2021;12(1):123. 33495437 10.1038/s41419-020-03376-y PMC7835218

[44] Naghavi L, Schwalbe M, Ghanem A, Naumann M. Deubiquitinylase USP47 promotes RelA phosphorylation and survival in gastric cancer cells. Biomedicine. 2018;6(2):62. 10.3390/biomedicines6020062 PMC6027160 29786670

[45] Bufalieri F, Infante P, Bernardi F, Caimano M, Romania P, Moretti M, Lospinoso Severini L, Talbot J, Melaiu O, Tanori M, et al. ERAP1 promotes hedgehog-dependent tumorigenesis by controlling USP47-mediated degradation of βTrCP. Nat Commun. 2019;10(1):3304. 31341163 10.1038/s41467-019-11093-0 PMC6656771

[46] Huang X, Shen F, Wang D, Wen Y, Zhang Y, Sheng N, Jiang X, Wu S, Mi Y. M2pep-modified liposomal nanoparticles delivering siITGB4 induce apoptosis and inhibit NSCLC metastasis via macrophage reprogramming. Apoptosis. 2026;31(3):78. 41774227 10.1007/s10495-025-02243-5

[47] Liu Y, Xu B, Zhao T, Liu H, Pan Y, Zhao S, Chen Q, Wang L, Bai G, Zhang N, et al. TAF15 in tumor-associated macrophages enhances protumorigenic polarization and promotes cholangiocarcinoma progression. JHEP Rep. 2025;7(12): Article 101545. 41244301 10.1016/j.jhepr.2025.101545 PMC12615741

[48] Bikfalvi A, da Costa CA, Avril T, Barnier JV, Bauchet L, Brisson L, Cartron PF, Castel H, Chevet E, Chneiweiss H, et al. Challenges in glioblastoma research: Focus on the tumor microenvironment. Trends Cancer. 2023;9(1):9–27. 36400694 10.1016/j.trecan.2022.09.005

[49] Theotoka D, Liu Z, Wall S, Galor A, Al Bayyat GJ, Feuer W, Jianhua W, Karp CL. Optical coherence tomography angiography in the evaluation of vascular patterns of ocular surface squamous neoplasia during topical medical treatment. Ocul Surf. 2022;25:8–18. 35358712 10.1016/j.jtos.2022.03.006 PMC9378455

[50] Shen R, Peng L, Zhou W, Wang D, Jiang Q, Ji J, Hu F, Yuan H. Anti-angiogenic nano-delivery system promotes tumor vascular normalizing and micro-environment reprogramming in solid tumor. J Control Release Off J Control Release Soc. 2022;349:550–564. 10.1016/j.jconrel.2022.07.015 35841997

[51] He G, Peng X, Wei S, Yang S, Li X, Huang M, Tang S, Jin H, Liu J, Zhang S, et al. Exosomes in the hypoxic TME: From release, uptake and biofunctions to clinical applications. Mol Cancer. 2022;21(1):19. 35039054 10.1186/s12943-021-01440-5 PMC8762953

[52] Xue Z, Liu J, Xing W, Mu F, Wu Y, Zhao J, Liu X, Wang D, Wang J, Li X, et al. Hypoxic glioma-derived exosomal miR-25-3p promotes macrophage M2 polarization by activating the PI3K-AKT-mTOR signaling pathway. J Nanobiotechnol. 2024;22(1):628. 10.1186/s12951-024-02888-5 PMC11481566 39407269

[53] Wu B, Shi X, Jiang M, Liu H. Cross-talk between cancer stem cells and immune cells: Potential therapeutic targets in the tumor immune microenvironment. Mol Cancer. 2023;22(1):38. 36810098 10.1186/s12943-023-01748-4 PMC9942413

[54] Da Ros M, De Gregorio V, Iorio AL, Giunti L, Guidi M, De Martino M, Genitori L, Sardi I. Glioblastoma chemoresistance: The double play by microenvironment and blood-brain barrier. Int J Mol Sci. 2018;19(10):2879. 30248992 10.3390/ijms19102879 PMC6213072

[55] Tang F, Zhang JN, Xu LY, Zhao XL, Wan F, Ao H, Peng C. Endothelial-derived exosomes: A novel therapeutic strategy for LPS-induced myocardial damage with anisodamine. Int J Biol Macromol. 2024;282(Pt 4): Article 136993. 39489255 10.1016/j.ijbiomac.2024.136993

[56] Zhang H, Zhao Y, Zhang Y, Hang R, Yao X, Hang R. Exosomes derived from macrophages upon cobalt ion stimulation promote angiogenesis. Colloids Surf B Biointerfaces. 2021;203: Article 111742. 33838581 10.1016/j.colsurfb.2021.111742

[57] Gupta M, Levine SR, Spitale RC. Probing nascent RNA with metabolic incorporation of modified nucleosides. Acc Chem Res. 2022;55(18):2647–2659. 36073807 10.1021/acs.accounts.2c00347

[58] Misir S, Aljabali AA, Yaman SÖ, Petrović N, Obeid MA. Small non-coding RNAs as therapeutic targets with delivery strategies in cancer treatment and their clinical applications. Int J Pharm. 2025;685: Article 126231. 41052743 10.1016/j.ijpharm.2025.126231

[59] Deforzh E, Kharel P, Zhang Y, Karelin A, El Khayari A, Ivanov P, Krichevsky AM. HOXDeRNA activates a cancerous transcription program and super enhancers via genome-wide binding. Mol Cell. 2024;84(20):3950–3966. 39383879 10.1016/j.molcel.2024.09.018 PMC11490371

[60] Chen Y, Liu Y, Xiong J, Ouyang L, Tang M, Mao C, Li L, Xiao D, Liu S, Yang Z, et al. LINC02774 inhibits glycolysis in glioma to destabilize HIF-1α dependent on transcription factor RP58. MedComm. 2023;4(5): Article e364. 37701531 10.1002/mco2.364 PMC10494996

[61] Leisegang MS, Bains JK, Seredinski S, Oo JA, Krause NM, Kuo CC, Günther S, Sentürk Cetin N, Warwick T, Cao C, et al. HIF1α-AS1 is a DNA:DNA:RNA triplex-forming lncRNA interacting with the HUSH complex. Nat Commun. 2022;13(1):6563. 36323673 10.1038/s41467-022-34252-2 PMC9630315

[62] Su YY, Ji FY, Zheng ZJ, Peng JY, Xie JJ. The role of super-enhancer-driven lncRNAs in cancer. Comput Struct Biotechnol J. 2025;27:3897–3907. 41542082 10.1016/j.csbj.2025.09.011 PMC12799938

[63] Deforzh E, Uhlmann EJ, Das E, Galitsyna A, Arora R, Saravanan H, Rabinovsky R, Wirawan AD, Teplyuk NM, El Fatimy R, et al. Promoter and enhancer RNAs regulate chromatin reorganization and activation of miR-10b/HOXD locus, and neoplastic transformation in glioma. Mol Cell. 2022;82(10):1894–1908. 35390275 10.1016/j.molcel.2022.03.018 PMC9271318

[64] Xie Y, Chen Y, Li Z, Zhu J, Liu M, Zhang Y, Dong Z. Enhancer transcription detected in the nascent transcriptomic landscape of bread wheat. Genome Biol. 2022;23(1):109. 35501845 10.1186/s13059-022-02675-1 PMC9063354

[65] Zhu Y, Gujar AD, Wong CH, Tjong H, Ngan CY, Gong L, Chen YA, Kim H, Liu J, Li M, et al. Oncogenic extrachromosomal DNA functions as mobile enhancers to globally amplify chromosomal transcription. Cancer Cell. 2021;39(5):694–707. 33836152 10.1016/j.ccell.2021.03.006 PMC8119378

[66] Gui Y, Qin H, Zhang X, Chen Q, Ye F, Tian G, Yang S, Ye Y, Pan D, Zhou J, et al. Glioma-astrocyte connexin43 confers temozolomide resistance through activation of the E2F1/ERCC1 axis. Neuro-Oncology. 2025;27(3):711–726. 39514365 10.1093/neuonc/noae237 PMC11889727

[67] Matsubayashi T, Yoshioka K, Mon SS, Katsuyama M, Jia C, Yamaguchi T, Hara RI, Nagata T, Nakagawa O, Obika S, et al. Favorable efficacy and reduced acute neurotoxicity by antisense oligonucleotides with 2′,4′-BNA/LNA with 9-(aminoethoxy)phenoxazine. Mol Ther Nucleic Acids. 2024;35(2): Article 102161. 38978695 10.1016/j.omtn.2024.102161 PMC11229412

[68] Lauffer MC, van Roon-Mom W, Aartsma-Rus A. Possibilities and limitations of antisense oligonucleotide therapies for the treatment of monogenic disorders. Commun Med. 2024;4(1):6. 38182878 10.1038/s43856-023-00419-1 PMC10770028

[69] Chen J, Sheng CW, Peng Y, Wang K, Jiao Y, Palli SR, Cao H. Transcript level and sequence matching are key determinants of off-target effects in RNAi. J Agric Food Chem. 2024;72(1):577–589. 38135672 10.1021/acs.jafc.3c07434

[70] Zhao J, Li H, Zhao S, Wang E, Zhu J, Feng D, Zhu Y, Dou W, Fan Q, Hu J, et al. Epigenetic silencing of miR-144/451a cluster contributes to HCC progression via paracrine HGF/MIF-mediated TAM remodeling. Mol Cancer. 2021;20(1):46. 33658044 10.1186/s12943-021-01343-5 PMC7927270

[71] Yang Y, Mei H, Han X, Zhang X, Cheng J, Zhang Z, Wang H, Xu H. Synthetic CRISPR/dCas9-KRAB system driven by specific PSA promoter suppresses malignant biological behavior of prostate cancer cells through negative feedback inhibition of PSA expression. Cell Mol Biol Lett. 2023;28(1):96. 38017385 10.1186/s11658-023-00508-y PMC10685504

[72] Wang B, Liu X, Li Z, Zeng K, Guo J, Xin T, Zhang Z, Li JF, Yang X. A nuclease-dead Cas9-derived tool represses target gene expression. Plant Physiol. 2024;195(3):1880–1892. 38478589 10.1093/plphys/kiae149

