# Supplementary material for: Hypoxic Endothelial-Cell-Derived Exosomal lnc-DKK3 Reprograms Tumor-Associated Macrophages via USP47/PD-L1/RelA Axis to Promote Glioma Progression
Source: Research (Wash D C). 2026 Sep 14;9:1431. doi: 10.34133/research.1431 (PMC13572801; doi:10.34133/research.1431)
Supplement: Supplementary file 1 [file research.1431.f1.zip › Figures S1-S6.pdf]

S1

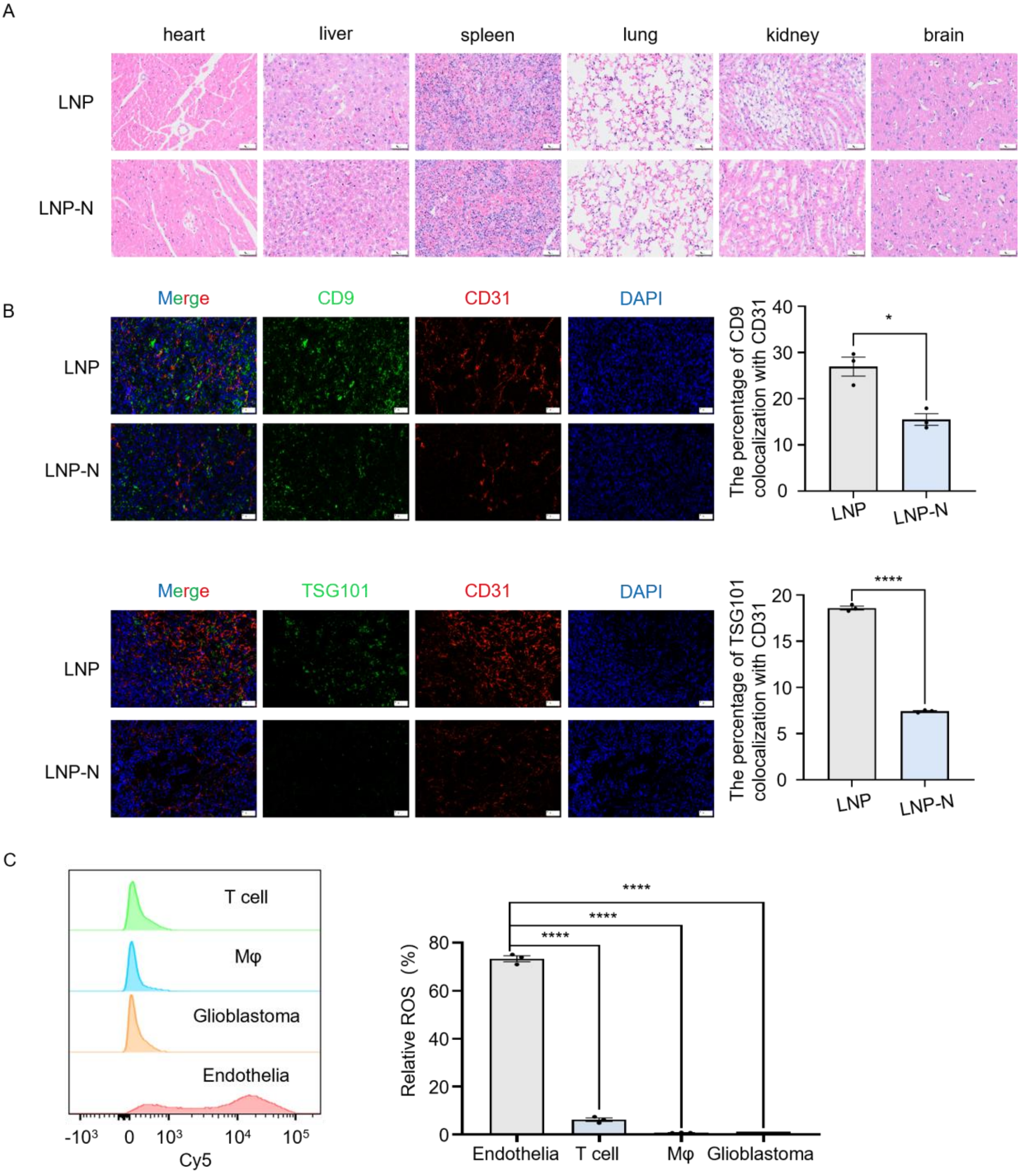
A
heart
liver
spleen
lung
kidney
brain
LNP
LNP-N
B
Merge
CD9
CD31
DAPI
LNP
LNP-N
The percentage of CD9 colocalization with CD31
0
10
20
30
40
*
LNP
LNP-N
Merge
TSG101
CD31
DAPI
LNP
LNP-N
The percentage of TSG101 colocalization with CD31
0
5
10
15
20
****
LNP
LNP-N
C
T cell
Mφ
Glioblastoma
Endothelia
-10³
0
10³
10⁴
10⁵
Cy5
Relative ROS (%)
0
20
40
60
80
****
****
****
Endothelia
T cell
Mφ
Glioblastoma

S2

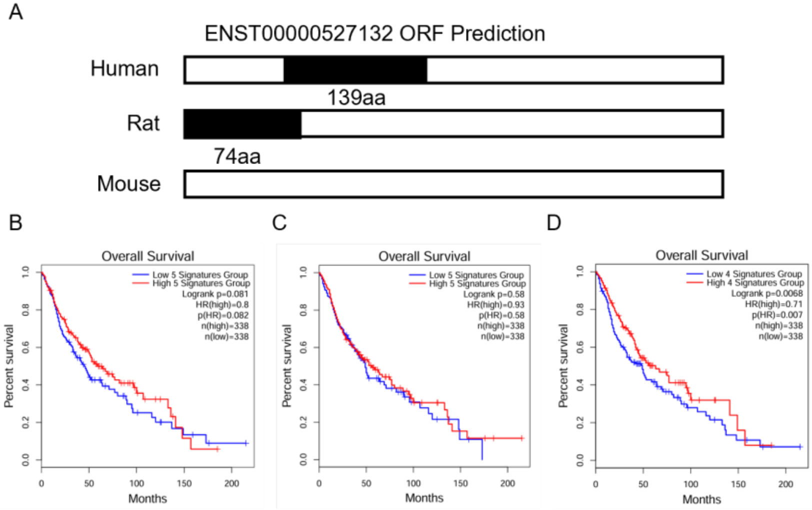


CCR7+LEF1+TCF7
+SELL+USP47

FOXP3+CTLA4+CCR8+
TNFRSF9+USP47

CD19+CD21+
CD22+USP47

S3

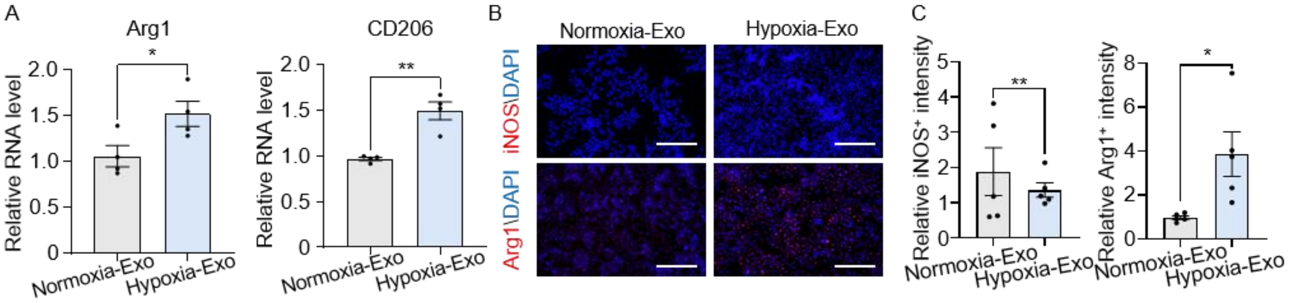
A
Arg1
*
Relative RNA level
2.0
1.5
1.0
0.5
0
Normoxia-Exo
Hypoxia-Exo
CD206
**
Relative RNA level
2.0
1.5
1.0
0.5
0
Normoxia-Exo
Hypoxia-Exo
B
Normoxia-Exo
Hypoxia-Exo
iNOS\DAPI
Arg1\DAPI
C
Relative iNOS+ intensity
5
4
3
2
1
0
**
Normoxia-Exo
Hypoxia-Exo
Relative Arg1+ intensity
8
6
4
2
0
*
Normoxia-Exo
Hypoxia-Exo

S4

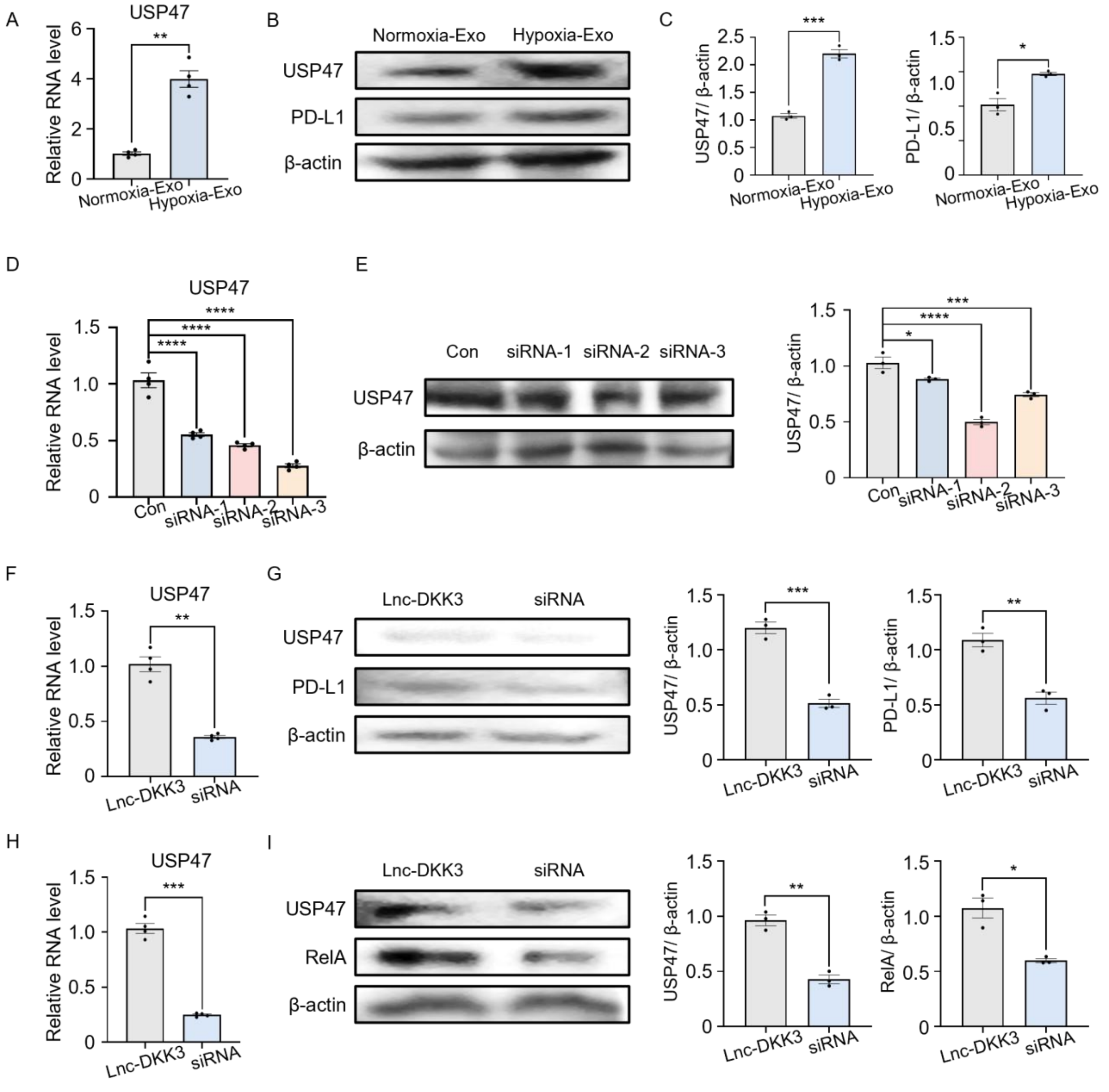
A
USP47
Relative RNA level
**
Normoxia-Exo
Hypoxia-Exo
B
Normoxia-Exo
Hypoxia-Exo
USP47
PD-L1
β-actin
C
***
USP47/ β-actin
Normoxia-Exo
Hypoxia-Exo
*
PD-L1/ β-actin
Normoxia-Exo
Hypoxia-Exo
D
USP47
Relative RNA level
****
Con
siRNA-1
siRNA-2
siRNA-3
E
Con
siRNA-1
siRNA-2
siRNA-3
USP47
β-actin
USP47/ β-actin
***
****
*
Con
siRNA-1
siRNA-2
siRNA-3
F
USP47
Relative RNA level
**
Lnc-DKK3
siRNA
G
Lnc-DKK3
siRNA
USP47
PD-L1
β-actin
USP47/ β-actin
***
PD-L1/ β-actin
**
Lnc-DKK3
siRNA
H
USP47
Relative RNA level
***
Lnc-DKK3
siRNA
I
Lnc-DKK3
siRNA
USP47
RelA
β-actin
USP47/ β-actin
**
RelA/ β-actin
*
Lnc-DKK3
siRNA

S5

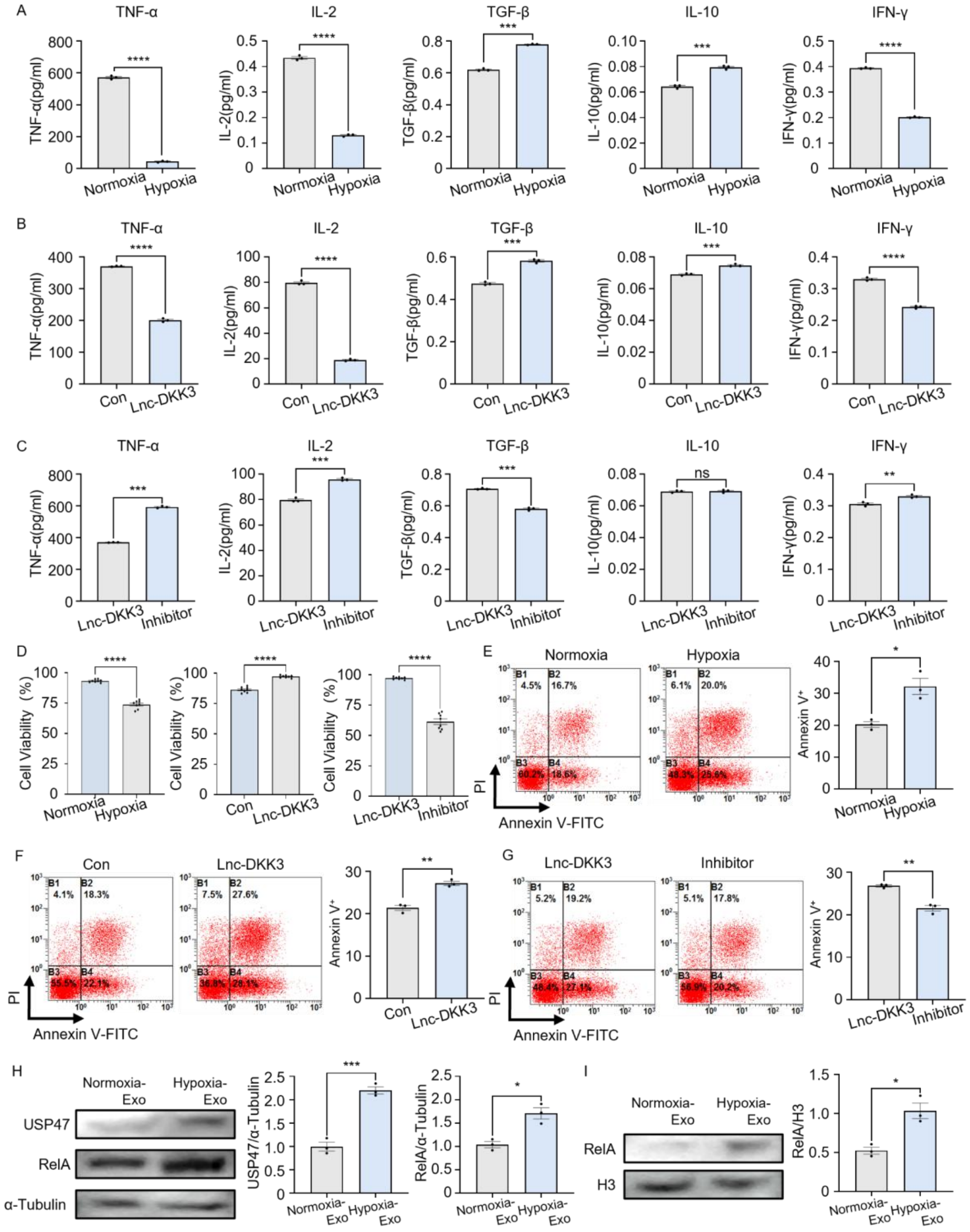
A
TNF-α
IL-2
TGF-β
IL-10
IFN-γ
TNF-α(pg/ml)
IL-2(pg/ml)
TGF-β(pg/ml)
IL-10(pg/ml)
IFN-γ(pg/ml)
Normoxia
Hypoxia
B
Con
Lnc-DKK3
C
Inhibitor
ns
D
Cell Viability (%)
E
Normoxia
Hypoxia
B1 4.5% B2 16.7%
B3 60.2% B4 18.6%
B1 6.1% B2 20.0%
B3 48.3% B4 25.6%
PI
Annexin V-FITC
Annexin V+
F
Con
Lnc-DKK3
B1 4.1% B2 18.3%
B3 55.5% B4 22.1%
B1 7.5% B2 27.6%
B3 36.8% B4 28.1%
G
Lnc-DKK3
Inhibitor
B1 5.2% B2 19.2%
B3 48.4% B4 27.1%
B1 5.1% B2 17.8%
B3 56.9% B4 20.2%
H
Normoxia-Exo
Hypoxia-Exo
USP47
RelA
α-Tubulin
USP47/α-Tubulin
RelA/α-Tubulin
I
RelA
H3
RelA/H3

S6

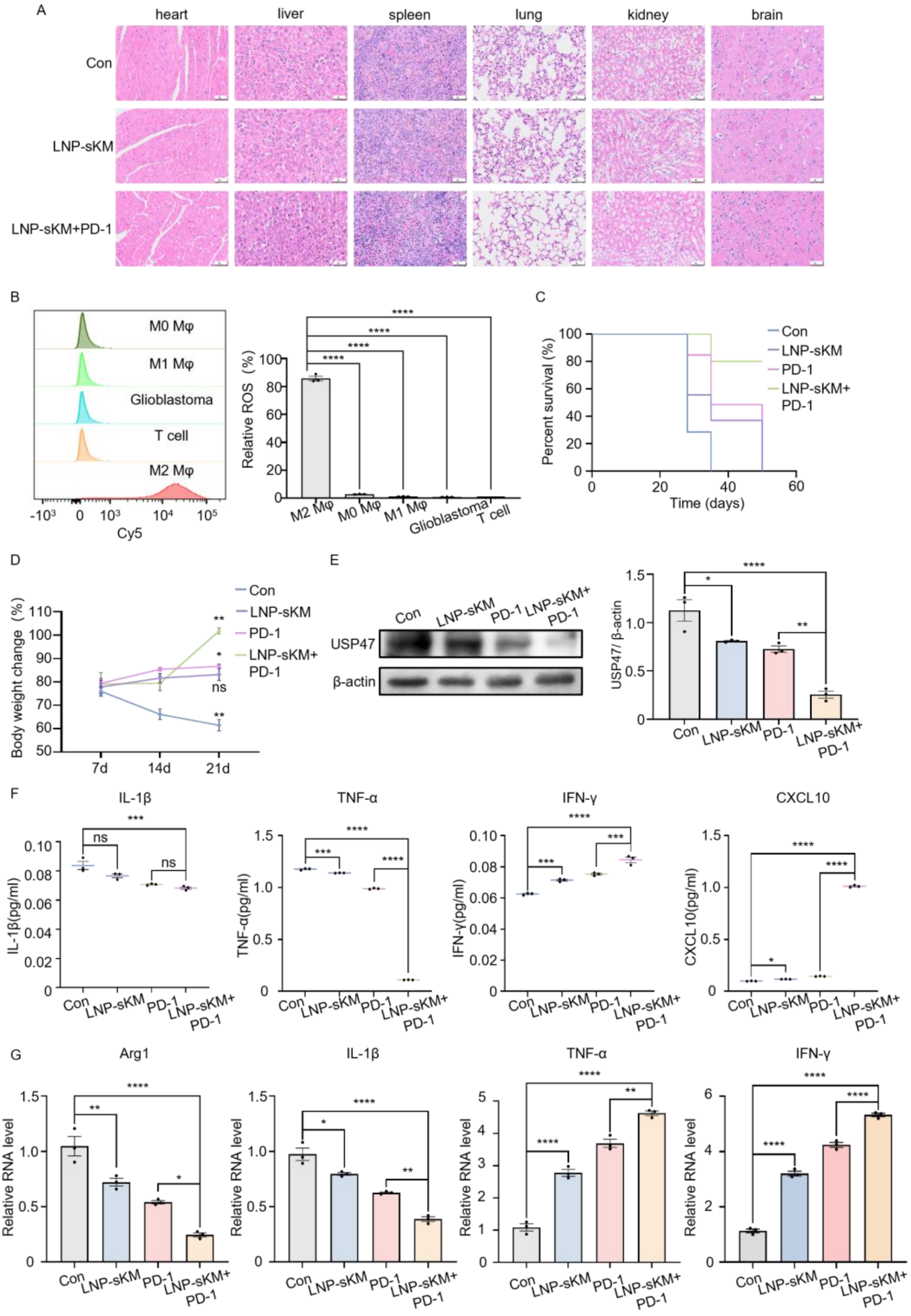
A
heart
liver
spleen
lung
kidney
brain
Con
LNP-sKM
LNP-sKM+PD-1
B
M0 Mφ
M1 Mφ
Glioblastoma
T cell
M2 Mφ
-10³
0
10³
10⁴
10⁵
Cy5
Relative ROS (%)
****
M2 Mφ
M0 Mφ
M1 Mφ
Glioblastoma
T cell
C
Percent survival (%)
Time (days)
Con
LNP-sKM
PD-1
LNP-sKM+ PD-1
D
Body weight change (%)
7d
14d
21d
ns
Con
LNP-sKM
PD-1
LNP-sKM+ PD-1
E
Con
LNP-sKM
PD-1
LNP-sKM+ PD-1
USP47
β-actin
USP47/ β-actin
F
IL-1β
IL-1β(pg/ml)
TNF-α
TNF-α(pg/ml)
IFN-γ
IFN-γ(pg/ml)
CXCL10
CXCL10(pg/ml)
ns
G
Arg1
IL-1β
TNF-α
IFN-γ
Relative RNA level
