# Supplementary material for: Hypoxic Endothelial-Cell-Derived Exosomal lnc-DKK3 Reprograms Tumor-Associated Macrophages via USP47/PD-L1/RelA Axis to Promote Glioma Progression
Source: Research (Wash D C). 2026 Sep 14;9:1431. doi: 10.34133/research.1431 (PMC13572801; doi:10.34133/research.1431)
Supplement: Supplementary file 1 [file research.1431.f1.zip › Materials and Methods for research.docx]

**Supplementary Information for**

**Hypoxic Endothelial Cell-Derived Exosomal lnc-DKK3 Reprograms Tumor-Associated Macrophages via USP47/PD-L1/RelA Axis to Promote Glioma Progression**

Zanhan Wang^#1^, Yongbo Zhao^#2^, Fan Fan^#3^, Chenchen Ji^#1^, Yamiao Chen^1^, Penghui Li^4^, Ang Ji^1^, Wei Bai^1^, Hongqing Chen^1^, Yunchao Yuan^1^, Juan Li^1^, Xiuli Cao^3^, Xia Li^*1^, Jifeng Liu^*5^, Junlong Zhao^*3^, Sanzhong Li^*1^

^#^These authors contributed equally

^*^To whom correspondence should be addressed:

Sanzhong Li, Email: sunny_3c@126.com; Junlong Zhao, Email: zhaojl@fmmu.edu.cn; Jifeng Liu, Email: 729122921@qq.com; Xia Li, Email: lixia_fmmu@126.com.

Department of Neurosurgery, Xijing Hospital, Air Force Medical University, Xi’an, China. Tel: 86-029-84775567;

**This PDF file includes:**

Material and methods

Legends for Figures S1-S6

**Material and methods**

**Mice and Cell lines**

**Cell Culture and Hypoxic Treatment**

The mouse glioma cell line GL261 and immortalized bone marrow-derived macrophages (iBMDM) were cultured in high-glucose Dulbecco’s Modified Eagle Medium (DMEM; Gibco) supplemented with 10% fetal bovine serum (FBS; Gibco) and 1% penicillin/streptomycin. Human umbilical vein endothelial cells (HUVECs; ATCC) were maintained in Endothelial Cell Medium (ECM; ScienCell) containing 5% FBS, 1% Endothelial Cell Growth Supplement (ECGS), and 1% penicillin/streptomycin. All cells were incubated at 37℃ in a humidified atmosphere with 5% CO2.

To mimic the hypoxic tumor microenvironment, cells were placed in a tri-gas incubator (Thermo Scientific) under conditions of 1% O2, 5% CO2, and 94% N2 for 24-72 hours. Normoxic controls were maintained at 21% O2. For conditioned medium (CM) collection, HUVECs were cultured under normoxic or hypoxic conditions for 48 hours. The supernatant was then collected, filtered through a 0.22μm membrane to remove cellular debris, and either used immediately for co-culture experiments or stored at -80℃.

**Detailed bioinformatic workflow including databases used, analysis parameters, and statistical methods**

This RNA-seq bioinformatics pipeline adopted standardized software, databases and unified statistical thresholds. Raw reads were quality-filtered with fastp v0.18.0, then rRNA sequences were eliminated via Bowtie2 v2.2.8. Clean reads were mapped to the reference genome using HISAT2 v2.1.0 with RF strand-specific mode. StringTie v1.3.4 assembled transcripts and calculated FPKM values; Cuffcompare screened novel transcripts, while CNCI and CPC jointly distinguished lncRNAs categorized by genomic positions. Pearson correlation and PCA (gmodels R package assessed sample consistency, and DESeq2/edgeR identified differentially expressed genes. GO, KEGG, DO and Reactome enrichments adopted hypergeometric tests (FDR ≤0.05), and GSEA utilized MSigDB. STRING v10 and Cytoscape v3.7.1 constructed PPI networks. Additional analyses included miRBase/miRPara miRNA prediction, Cufflinks UTR optimization, GATK/ANNOVAR variant filtering, and rMATS splicing detection. RNAplex predicted antisense lncRNA-mRNA pairs; cis targets were defined within ±100 kb, and trans targets were screened via strict Pearson correlation or WGCNA for subsequent functional enrichment.

**Exosome isolation and characterization protocol**

Serum samples underwent sequential centrifugation at 1500g for 10 min, 10,000g for 30 min, 0.22 μm filtration, and 100,000g for 1 h, with an additional PBS wash at 100,000g for 1 h. Cell culture supernatants and urine were processed at 10,000g for 30 min, concentrated via 100 kDa ultrafiltration at 3500×g for 15 min, filtered through 0.22 μm membranes, and ultracentrifuged at 120,000×g for 90 min. Animal tissues were minced in RPMI-1640, digested with collagenase IV and DNase I at 37°C with 500 rpm shaking, sequentially centrifuged at 300g for 10 min, 2000g for 20 min, 10,000g for 45 min, and finally 120,000×g for 70 min. Plant-derived nanovesicles were obtained by grinding, centrifugation at 3000g for 30 min, 10,000g for 60 min, 150,000g for 90 min, followed by sucrose gradient ultracentrifugation (8%, 15%, 30%, 45%, 60%) at 150,000g for 120 min, collecting the 30%/45% interface. Milk exosomes were isolated by centrifugation at 5000g for 10 min to collect whey, then 16,500g for 60 min, 0.22 μm filtration, and 100,000g for 90 min, with an additional 100,000g wash for 1 h.

Nanoparticle tracking analysis (NTA) was performed using a ZetaView_Particle Metrix PMX-120 instrument (Germany) calibrated with 100 nm polystyrene beads (Thermo Fisher). Samples were diluted in 1× PBS and analyzed under the following parameters: laser 5/40 mW 488 nm, sample pressure 1.0–1.1 KPa, SS decay 10%, threshold/sub settings 71.2–73.7/9.2–13.9, and minimum width 0.3 ms. The CY (normoxia) sample showed a particle concentration of 1.8E+8 particles/mL (dilution 32,000×, original 5.8E+12 particles/mL) with median diameter 97.1 nm; the QY (hypoxia) sample showed 1.7E+8 particles/mL (dilution 16,000×, original 2.7E+12 particles/mL) with median diameter 100.6 nm.

Transmission electron microscopy (TEM) analysis utilized negative staining with 2% phosphotungstic acid on 200-mesh formvar-coated copper grids. Samples (10 μL) were adsorbed onto grids for 10–15 min, stained for 3–5 min, air-dried, and visualized using an HT7700 transmission electron microscope (Hitachi, Japan). Both Normoxia and Normoxia samples exhibited typical cup-shaped exosome morphology.

Western blot validation was performed with SDS-PAGE on 10% polyacrylamide gels, transferred to PVDF membranes (0.2 or 0.45 μm), blocked with 5% non-fat milk, and incubated with primary antibodies overnight at 4°C: anti-CD9 (DLM, DA19027), anti-CD81 (Proteintech, 66866-1-Ig), anti-TSG101 (DLM, DA5789), and anti-Calnexin (Proteintech, 10427-2-AP). HRP-conjugated secondary antibodies (Proteintech) were incubated for 30 min at 37°C, and signals were visualized using ECL reagents (ABclonal or Vazyme) with X-ray film exposure. Both exosome samples (Exo-1 and Exo-2) were positive for CD9, CD81, and TSG101, while Calnexin was undetectable, confirming exosome purity without cellular contamination.

Nano-flow cytometry (NanoFCM) was additionally employed for surface marker detection. Exosomes (30 μL diluted to 90 μL) were incubated with FITC-conjugated anti-CD9, anti-CD63, and anti-CD81 antibodies (BD) at 37°C for 30 min, washed twice with PBS by ultracentrifugation at 110,000×g for 70 min at 4°C, and resuspended in 50 μL PBS for NanoFCM N30E analysis. normoxia sample showed positive rates of 22.4% (CD9), 17.5% (CD63), and 17.5% (CD81), with 0.3% blank control; Hypoxia showed 19.0% (CD9), 21.7% (CD63), and 24.2% (CD81), with 0.8% blank control.

**In Vitro Functional Assays.**

**Cell Proliferation and Clonogenicity:** Cell viability was assessed using the Cell Counting Kit-8 (CCK-8; Biosharp) according to the manufacturer’s instructions. For clonogenic assays, 1000 single cells were seeded per well in 6-well plates, cultured for 10–14 days, fixed with methanol, and stained with 0.1% crystal violet. Colonies containing >50 cells were counted.

**Wound Healing Assay:** Cells were seeded at high density in 12-well plates and grown to confluence. A sterile 200-μL pipette tip was used to create a uniform scratch. After washing with PBS to remove detached cells, fresh medium containing 1% FBS was added. Images of the scratch were captured at 0, 12, and 24 hours using an inverted microscope (Thermo M7000). The wound closure area was measured, and the migration rate was calculated using ImageJ.

**Macrophage Polarization:** iBMDM were stimulated with IFN-γ (20 ng/mL; PeproTech) plus LPS (50 ng/mL; Sigma) to induce M1 polarization, or with IL-4 (20 ng/mL; PeproTech) to induce M2 polarization for 24 hours. Alternatively, iBMDMs were transfected with an lnc-DKK3 overexpression plasmid. Polarization status was evaluated by qRT-PCR and flow cytometry analysis of M1 (e.g., iNOS, CD86) and M2 (e.g., Arg1, CD206) markers.

**Molecular Analyses.**

**Quantitative Real-Time PCR (qRT-PCR):** Total RNA was extracted with TRIzol reagent (Invitrogen), reverse-transcribed into cDNA, and amplified using SYBR Green Premix (Takara) on a QuantStudio 5 Real-Time PCR System (Applied Biosystems). Gene expression was normalized to Gapdh and calculated using the 2^(-△△Ct) method. Targets included lnc-DKK3, USP47, M2 macrophage markers, glioma stemness genes, and T-cell exhaustion markers.

**Western Blotting:** Proteins were extracted with RIPA lysis buffer, quantified by BCA assay, separated by SDS-PAGE, and transferred to PVDF membranes. After blocking with 5% non-fat milk, membranes were incubated overnight at 4℃ with primary antibodies against USP47, PD-L1, RelA, α-Tubulin, H3(Cell Signaling Technology), and others, followed by incubation with HRP-conjugated secondary antibodies. Signals were detected using an ECL substrate.

**Immunofluorescence Staining:** Cells or frozen tissue sections were fixed, permeabilized, blocked, and incubated with primary antibodies overnight at 4℃, followed by Alexa Fluor-conjugated secondary antibodies (Invitrogen) and DAPI counterstaining. Images were acquired using a confocal microscope (Thermo M5000).

**Isolation, purification and activation of primary mouse T cells**

Spleens from 8–12-week-old C57BL/6 mice were mechanically ground to prepare single-cell suspensions. Primary T lymphocytes were purified through magnetic bead negative selection kits to eliminate B cells, myeloid cells and other miscellaneous cell populations. The obtained pure CD3+ T cells were activated with CD3/CD28 stimulation beads, then co-cultured with GL261 glioma cells subjected to different treatments.

**Co-culture of GL261 glioma cells and T cells**

Co-culture experiments were performed using GL261 murine glioma cells, subjected to various preconditioning treatments, as target cells, which were then co-cultured with primary murine CD3⁺ T lymphocytes pre-activated with anti-CD3/CD28 antibodies for 24 h. Effector T cells and target glioma cells were seeded at a ratio of 10:1 in RPMI 1640 complete medium supplemented with 10% fetal bovine serum, 50 μM β-mercaptoethanol, 1 mM sodium pyruvate, and 2 mM L-glutamine, and incubated at 37°C with 5% CO_2_ for 24 h. After co-culture, suspended T cells were harvested for qRT‑PCR analysis of exhaustion-related gene expression, and culture supernatants were collected for ELISA. For preconditioning, GL261 cells were treated for 24 h with hypoxic HUVEC-conditioned medium (prepared by culturing HUVECs under 1% O_2_ for 48 h, collecting the supernatant, and filtering through a 0.22‑μm membrane) diluted 1:1 with DMEM, or with 10 μM USP47 inhibitor for the entire 24‑h preconditioning period. Prior to co-culture, adherent GL261 cells were washed twice with PBS to remove residual preconditioning factors, ensuring that the observed effects originated solely from the altered tumor cell status. All experiments were performed with at least three independent biological replicates, and data are presented as mean ± SD, with intergroup comparisons evaluated by one‑way analysis of variance.

**Dual-Luciferase Reporter Assay**

Reporter plasmid GV148-enhancer-Luc: The full-length USP47 super-enhancer fragment with H3K4me1/H3K27ac active epigenetic marks was amplified and inserted into the GV148 luciferase backbone to drive firefly luciferase expression. lnc-DKK3 overexpression plasmid GV172-lnc-DKK3: The full transcript sequence of lnc-DKK3 (ENST00000527132) was cloned into the GV172 overexpression vector; empty pcDNA3.1 was used as negative control (pcDNA3.1-NC). Internal reference plasmid pRL-TK (abbreviated TK): Carries Renilla luciferase, co-transfected in all groups to normalize transfection efficiency and eliminate well-to-well variation.

Cells were seeded in 24-well culture plates and transfected at 70%–80% confluence using liposome transfection reagent. At 48 h post-transfection, culture medium was discarded, and cells were lysed using cell lysis buffer supplied with the dual-luciferase detection kit. Firefly luciferase activity and Renilla luciferase activity were sequentially measured via a microplate luminometer. The relative luciferase activity of each well was calculated as firefly luminescence value / Renilla luminescence value. Each group contained at least three independent biological replicates. One-way analysis of variance (ANOVA) followed by Tukey’s multiple comparison test was used to evaluate statistical differences among groups.

**CRISPRi system**

The CRISPRi system was constructed with a dual‑sgRNA cassette targeting the mouse lnc‑DKK3 locus, using sgRNA sequences sgRNA‑1 (GTTAACTACAGATCAAGCAG) and sgRNA‑2 (TAAAGACTAGCACAAAGAAC), cloned into the GV469 backbone (MCS‑SV40‑neomycin) at MluI/HindIII sites, with the non‑targeting control U6‑sg(NC) assembled in the KV683 vector (MCS‑EF1α‑NLS‑MCP‑LSD1‑SV40‑neomycin). The dCas9‑KRAB effector was generated in the GV712 backbone (CMV enhancer‑MCS‑SV40‑puromycin) via XbaI cloning, producing a 4508‑bp insert encoding the full‑length dCas9‑KRAB fusion protein.

**Synthesis of LNPs functionalized with various targeting ligands.**

To inhibit the secretion of endothelial cell-derived exosomes, APRPG@Nexinhib20@LNP was prepared as follows. DLin-MC3-DMA, DSPC, cholesterol, DSPE-PEG2000-APRPG, and Nexinhib20 were dissolved in ethanol at a molar ratio of 40:10:35:10:5. The lipid mixture was rapidly injected into an aqueous buffer (50 mM citrate, pH4) under vigorous mixing to achieve a final ethanol concentration of 30% (v/v). The resulting dispersion was allowed to equilibrate at room temperature for 2 min prior to extrusion. Hydrated lipids were then extruded through two stacked polycarbonate membranes with 80-nm pores (Nuclepore) at 22 °C using a Lipex Extruder (Northern Lipids, Vancouver, Canada) until a homogeneous population of vesicles with a hydrodynamic diameter of 70-90 nm, confirmed by dynamic light scattering (DLS), was obtained.

For CRISPR interference (CRISPRi) in M2 macrophages, KRAB@M2pep@LNP was formulated using the preformed vesicle method. Preformed vesicles composed of DLin-MC3-DMA, DSPC, cholesterol, and DSPE-PEG2000-M2pep (molar ratio 40:10:40:10) were first prepared as described above (without drug or nucleic acid loading). An equal molar mixture of dCas9-KRAB and sgRNA plasmids, solubilized in 50 mM citrate buffer (pH4) containing 30% ethanol, was slowly added to the preformed vesicles under gentle stirring. The mixture was incubated at 35 °C for 30 min to facilitate vesicle reorganization and efficient plasmid encapsulation.

Both APRPG@Nexinhib20@LNP and KRAB@M2pep@LNP formulations were subsequently concentrated and purified by ultrafiltration to remove residual ethanol and exchange the buffer, and were stored at 4 °C for immediate use or further characterization.

**Statistical Analysis.**

All gene correlation and survival association analyses visualized in figures were performed using the GEPIA2 web server with TCGA glioma datasets. A consistent median expression cutoff rule was applied for all grouping strategies. Log-rank test was used to compare overall survival differences between cohorts.

All in vitro experiments were performed in at least three independent replicates. Data are presented as Mean ± SEM. Statistical analysis was performed using GraphPad Prism 9.0. Comparisons between two groups were analyzed by two-tailed unpaired Student’s t-test, and comparisons among multiple groups were analyzed by one-way analysis of variance (ANOVA). A p-value<0.05 was considered statistically significant.

**In Vivo Therapeutic Experiments.**

Tumor-bearing mice with different treatments were monitored weekly by bioluminescence imaging. Body weight and survival were recorded throughout the treatment period. All animal experiments were performed in accordance with protocols approved by the Animal Experiment Management Committee of the Fourth Military Medical University. Wild-type male C57BL/6 mice (8-12 weeks old) were housed under specific pathogen-free conditions. An orthotopic glioma model was established by stereotactically injecting luciferase-expressing GL261 cells (1×105 cells in 2μL PBS) into the right striatum (coordinates: 0.5mm posterior, 2.0mm lateral to the bregma, and 3.0mm deep). Tumor growth was monitored on days 7, 14, and 21 post-implantations using an in vivo imaging system (IVIS Spectrum; PerkinElmer). Mice were randomized into experimental groups based on bioluminescence signal intensity to ensure equivalent initial tumor burden. At the experimental endpoint, mice were perfused transcardially. Brain tissues were either fixed in 4% paraformaldehyde for paraffin embedding and sectioning or snap-frozen in liquid nitrogen for RNA extraction. Tumor area and fluorescence intensity were quantified using Image-Pro Plus or Image J software.

**Figure S1. Characterization of LNP-N safety, targeting specificity, and in vivo exosome blockade efficiency.**

(A) Toxicity assessment of LNP-N and control LNP formulations. HE staining of major organs (heart, liver, spleen, lung, kidney, and brain).

(B) Immunofluorescence analysis of brain tissue sections showing CD31⁺ endothelial cells (red) co-localized with exosomal markers CD9 (green) and TSG101 (green) in LNP-N-treated versus LNP-treated mice.

(C) In vitro cellular uptake assay demonstrating preferential internalization of fluorescently labeled LNP-N by endothelial cells (HUVECs) compared with immune cells (macrophages), T cell or glioblastoma (GL261).

**p* < 0.05, ***p* < 0.01, ****p* < 0.001, *****p* < 0.0001 two-tailed unpaired Student’s t-test or One-Way ANOVA; Mean ± SEM; Scale bar: 50 μm.

**Figure S2. lnc‑DKK3 ORF conservation and prognostic value of immune cell co‑expression signatures in glioma.**

(A) Open reading frame (ORF) prediction analysis demonstrates poor conservation of predicted ORFs for lnc-DKK3 across different species.

(B-D) Kaplan‑Meier analysis of TCGA glioma patients demonstrated that co‑expression of T cell (CCR7+LEF1+TCF7+SELL), Treg (FOXP3+CTLA4+CCR8+TNFRSF9) and B cell (CD19+CD21+CD22) signatures correlated with unfavorable survival

**Figure S3. Hypoxic endothelial cell‑derived exosomes promote M2‑like polarization of macrophages.**

Isolated exosomes from normoxic or hypoxic HUVEC supernatants were directly co‑cultured with iBMDMs for 24 h.

(A) qRT-PCR detection of macrophage polarization marker expression after co-culture with exosomes from normoxic or hypoxic endothelial cells.

(B-C) Immunofluorescence staining results and quantification.

**p* < 0.05, ***p* < 0.01, ****p* < 0.001, *****p* < 0.0001 two-tailed unpaired Student’s t-test; Mean ± SEM; Scale bar: 150 μm.

**Figure S4. USP47 is upregulated by hypoxic endothelial exosomes and serves as a downstream effector of lnc‑DKK3 in macrophages.**

(A) qRT‑PCR analysis of USP47 mRNA expression in iBMDMs following co‑culture with exosomes isolated from normoxic or hypoxic HUVEC supernatants.

(B) Representative Western blot images of PD‑L1 and USP47 protein expression in iBMDMs treated with normoxic or hypoxic exosomes.

(C) Quantification of PD‑L1 and USP47 protein levels relative to loading control, showing increased expression in hypoxic exosome‑treated cells.

(D) qRT‑PCR validation of USP47 knockdown efficiency in iBMDMs transfected

(E) Representative Western blot images and corresponding quantification demonstrating decreased USP47 and PD‑L1 protein levels following USP47 siRNA knockdown.

(F) qRT‑PCR analysis of USP47 mRNA in iBMDMs transfected with lnc‑DKK3 overexpression or in combination with siUSP47.

(G) Western blot analysis of USP47 and PD-L1 protein level in iBMDMs transfected with lnc‑DKK3 overexpression or in combination with siUSP47.

(H) qRT‑PCR analysis of USP47 mRNA in GL261 transfected with lnc‑DKK3 overexpression or in combination with siUSP47.

(I) Representative Western blot images and quantification.

**p* < 0.05, ***p* < 0.01, ****p* < 0.001, *****p* < 0.0001 two-tailed unpaired Student’s t-test or One-Way ANOVA; Mean ± SEM.

**Figure S5. USP47 is upregulated by hypoxic endothelial exosomes and mediates lnc‑DKK3‑induced RelA activation and T‑cell exhaustion in glioma cells.**

(A-C) ELISA quantification of T-cell exhaustion markers.

(D) CCK‑8 proliferation assay showing proliferative capacity of T cells.

(E-G) Flow cytometry‑based cytotoxicity assay and statistics.

(H-I) Western blot revealed increased RelA nuclear translocation upon hypoxic endothelial exosomes medium exposure.

**p* < 0.05, ***p* < 0.01, ****p* < 0.001, *****p* < 0.0001 two-tailed unpaired Student’s t-test; Mean ± SEM.

**Figure S6. Safety, targeting specificity, in vivo efficacy, and immune microenvironment remodeling by LNP‑sKM in the GL261 glioblastoma model.**

(A) Toxicity assessment of LNP‑sKM. Representative HE staining of major organs (heart, liver, spleen, lung, kidney, and brain) from mice.

(B) Targeting specificity of LNP‑sKM. Relative ROS levels in different cell types (M0, M1, and M2 macrophages, glioblastoma cells, and T cells) following incubation with fluorescently labeled LNP‑sKM. M2 macrophages exhibited the highest uptake, confirming M2pep‑mediated preferential targeting of M2‑like tumor‑associated macrophages.

(C) Kaplan‑Meier survival analysis of glioma‑bearing mice treated with Con, LNP‑sKM, anti‑PD‑1, or LNP‑sKM+PD‑1.

(D) Body weight changes in mice from each treatment group over the 21‑day observation period.

(E) Western blot analysis of USP47 protein levels in brain tumor tissues from different treatment groups.

(F) ELISA analysis of tumor tissue lysates.

(G) qRT‑PCR analysis of tumor tissues.

**p* < 0.05, ***p* < 0.01, ****p* < 0.001, *****p* < 0.0001 One-Way ANOVA; Mean ± SEM.
