# Supplementary material for: Hypoxic Endothelial-Cell-Derived Exosomal lnc-DKK3 Reprograms Tumor-Associated Macrophages via USP47/PD-L1/RelA Axis to Promote Glioma Progression
Source: Research (Wash D C). 2026 Sep 14;9:1431. doi: 10.34133/research.1431 (PMC13572801; doi:10.34133/research.1431)
Supplement: Supplementary file 1 [file research.1431.f1.zip › supplementary data.docx]

**Supplementary Table 1. Primers and oligonucleotides used in this study.**

| Gene | Purpose | Sequence |
| --- | --- | --- |
| ENST00000687392-F | qPCR | ACGTGTCTGCCTTCAACAA |
| ENST00000687392-R | qPCR | TCATTTCCACACTCCCTCAAC |
| ENST00000527132-F | qPCR | GCTGTGGGTAGATGTGCAATA |
| ENST00000527132-R | qPCR | CTGGGAAGAAGATGTAGGAAGAAG |
| ENST00000564984-F | qPCR | TGTCTTAGGGAGAGGGCATATC |
| ENST00000564984-R | qPCR | GGAGGACATCTATGCCAACATC |
| ENST00000555486-F | qPCR | CACATCTGTCCTCTGCTTCTC |
| ENST00000555486-R | qPCR | CCTTCTTGGTGGTGCCTATATT |
| ENST00000654392-F | qPCR | TCCTAGCCCTGAGTGATCTT |
| ENST00000654392-R | qPCR | TAGGTGGCACCAACACTATTC |
| iNOS-F | qPCR | GCAGAGATTGGAGGCCTTGTG |
| iNOS-R | qPCR | GGGTTGTTGCTGAACTTCCAGTC |
| MCP1-F | qPCR | GCTACAAGAGGATCACCAGCAG |
| MCP1-R | qPCR | GTCTGGACCCATTCCTTCTTGG |
| Arg1-F | qPCR | AGACAGCAGAGGAGGTGAAGAG |
| Arg1-R | qPCR | CGAAGCAAGCCAAGGTTAAAGC |
| MR-F | qPCR | GAGGGAAGCGAGAGATTATGGA |
| MR-R | qPCR | GCCTGATGCCAGGTTAAAGCA |
| OCT4-F | qPCR | CACCATCTGTCGCTTCGAGG |
| OCT4-R | qPCR | AGGGTCTCCGATTTGCATATCT |
| CD133-F | qPCR | CTGCGATAGCATCAGACCAAGC |
| CD133-R | qPCR | CTTTTGACGAGGCTCTCCAGATC |
| USP47-F | qPCR | AGAGACTGTCGCTGCTCCTGTA |
| USP47-R | qPCR | CCAGCACTATCCTCATGGAGTC |
| GZMB-F | qPCR | CAGGAGAAGACCCAGCAAGTCA |
| GZMB-R | qPCR | CTCACAGCTCTAGTCCTCTTGG |
| IL-2-F | qPCR | GCGGCATGTTCTGGATTTGACTC |
| IL-2-R | qPCR | CCACCACAGTTGCTGACTCATC |
| TIM3-F | qPCR | ACAGACACTGGTGACCCTCCAT |
| TIM3-R | qPCR | CAGCAGAGACTCCCACTCCAAT |
| TOX-F | qPCR | AAGATGGCGCACTGCTCTCCAA |
| TOX-R | qPCR | CATGCTTGCCTGCTGTCTGATG |
| PD-1-F | qPCR | CGGTTTCAAGGCATGGTCATTGG |
| PD-1-R | qPCR | TCAGAGTGTCGTCCTTGCTTCC |
| RELA-F | qPCR | TCCTGTTCGAGTCTCCATGCAG |
| RELA-R | qPCR | GGTCTCATAGGTCCTTTTGCGC |
| PD-L1-F | qPCR | TGCGGACTACAAGCGAATCACG |
| PD-L1-R | qPCR | CTCAGCTTCTGGATAACCCTCG |
| Actin-F | qPCR | CATTGCTGACAGGATGCAGAAGG |
| Actin-R | qPCR | TGCTGGAAGGTGGACAGTGAGG |
| LOC132412331-1 | gRNA | GTTAACTACAGATCAAGCAG |
| LOC132412331-2 | gRNA | TAAAGACTAGCACAAAGAAC |
| USP47 | siRNA | TGAAAAGGGATGTGCAAAA |
